# Biomimetic and Natural Exosomes in Drug Delivery: A Critical Review

**DOI:** 10.3390/biomimetics11100747

**Published:** 2026-10-16

**Authors:** Natalia Robles-Anda, Raquel González-Jabardo, Miguel Ladero, M. Esther Gallardo

**Affiliations:** 1 Translational Research with iPS Cells Group, Instituto de Investigación Sanitaria Hospital 12 de Octubre, imas12, Avda. de Córdoba s/n, 28041 Madrid, Spain; 2 Department of Chemical Engineering and Materials, University Complutense of Madrid, Avda. Complutense s/n, 28040 Madrid, Spain

**Keywords:** exosome, biomimetic, exosome-like, nanoparticle, nanoplatform, nanocarriers

## Abstract

Exosomes are biological nanoparticles (30–150 nm) secreted by most eukaryotic cells. They are composed of a lipid bilayer membrane and an inner compartment that stores various cellular components. In recent years, research on exosomes has grown exponentially due to their biocompatibility, low immunogenicity and cytotoxicity, and their permeability to biological barriers. Among the most promising applications of exosomes are their diagnostic and therapeutic potential, the latter being especially relevant due to their ability to act as a vehicle for drugs. Before exosomes can be applied clinically, it is necessary to optimize the isolation of these nanoparticles from various sources, as well as to carry out their physical and biochemical characterization. Furthermore, their ability to encapsulate molecules with therapeutic effects within them must be perfected. To this end, analytical methods are a key tool for developing each of these steps, and it is essential to understand their principles, strengths, and challenges. In addition, it is necessary to overcome limitations such as the availability of safe sources, the scalability of production, the standardization of isolation, purification, and encapsulation, the inherent heterogeneity of exosomes themselves, the prolongation of in vivo lifespan, and the long-term preservation of these nanoparticles. An alternative is the synthesis of exosome-like nanoparticles, which is less complex but more prone to high production and tailored structure and composition. This review addresses the latest advances in drug delivery focused on exosomes and exosome biomimetic nanoparticles, comparing their advantages and drawbacks from a practical perspective.

## 1. Introduction

### 1.1. Nanotechnology: Concept and Applications in Biomedicine

Nanotechnology is a branch of science that encompasses the synthesis, engineering, and utilization of materials ranging in size from 1 to 100 nm, known as nanomaterials. The synthesis of these materials has increased significantly in the last 40 years due to their wide range of applications in engineering, waste disposal, sporting goods, electronics, optics, clothing, food, cosmetics, and medicine. Nanotechnology is especially relevant in the medical field [1]. This is due to the ability to manipulate and adapt the physical chemistry of nanomaterials to the scale at which biomolecular interactions occur, allowing them to be used in the early detection of biomarkers, the specific targeting of cells and tissues, drug delivery systems, and the treatment of certain diseases [1]. However, the properties that make nanomaterials so attractive in medicine could also cause unintended harm (autoimmune reactions, heart, lung, and nervous system diseases, etc.). Therefore, the development of nanotoxicology is especially important to determine which properties of nanomaterials confer toxicity and should be avoided to develop safe materials, especially for medical purposes [1].

Within the field of nanotechnology, nanoparticles (NPs) are defined by the International Organization for Standardization (ISO) as nanoobjects with all external dimensions at the nanoscale (1–100 nm) [2]. NPs are characterized by their diverse shapes, sizes, and structures. They are also classified based on their composition into the following classes: organic (liposomes, micelles, protein complexes), carbon (fullerenes, carbon quantum dots), inorganic (metals, ceramics, semiconductors), and polymeric (natural or synthetic) [2]. Inorganic NPs have great potential for cancer diagnosis and therapy due to their enhanced light scattering and absorption properties. For example, gold NPs efficiently absorb light and convert it into localized heat, which can be exploited for selective photothermal cancer therapy. Similarly, ZnO and Fe_3_O_4_ nanoparticles have been used for targeted drug delivery and targeted tumor cell killing [2]. NPs have also been successfully employed in medical diagnostics to detect viruses or bacteria, as in the case of gold NPs conjugated to SARS-CoV-2 antigens to rapidly detect (in 10–15 min) the presence of SARS-CoV-2 IgM/IgA antibodies in blood samples [2].

### 1.2. Exosomes: Biological Nanoparticles

#### 1.2.1. Concept, Origin and Biogenesis

Exosomes are small, nanometric-sized (30–150 nm) extracellular vesicles (EVs) composed of a lipid bilayer and an internal compartment that stores various cellular components [3,4,5,6]. They are secreted by most eukaryotic cells and almost all cell types, including immune cells, stem cells, epithelial cells, endothelial cells, tumor cells, fibroblasts, nervous system cells, and platelets [4,6,7]. Furthermore, several studies have confirmed their presence in the intracellular space and in biological fluids such as plasma, amniotic, ascitic, and cerebrospinal fluid, urine, saliva, breast milk, bronchoalveolar lavage fluid, and even bile [4,6,7]. Under normal and pathological conditions, the kinetics and bioactivity of exosomes may differ [4,7]. For example, immature dendritic cells can increase their release when they interact with activated T cell clones or related CD4+ subpopulations [4,7]. Likewise, exosome production has also been controlled by p53-regulated transcription genes, such as tumor suppressor-activated pathway 6 (TSAP6), which increases under conditions of cellular stress [7]. In addition to cellular human exosomes (conventional exosomes), non-conventional exosomes can be found from animal products, bacteria, fungi, parasites and plants, and artificial exosomes designed in the laboratory that seek to mimic the properties of natural ones by specifically controlling their properties (biomimetic exosomes) [3,5,6].

**Figure 1:**
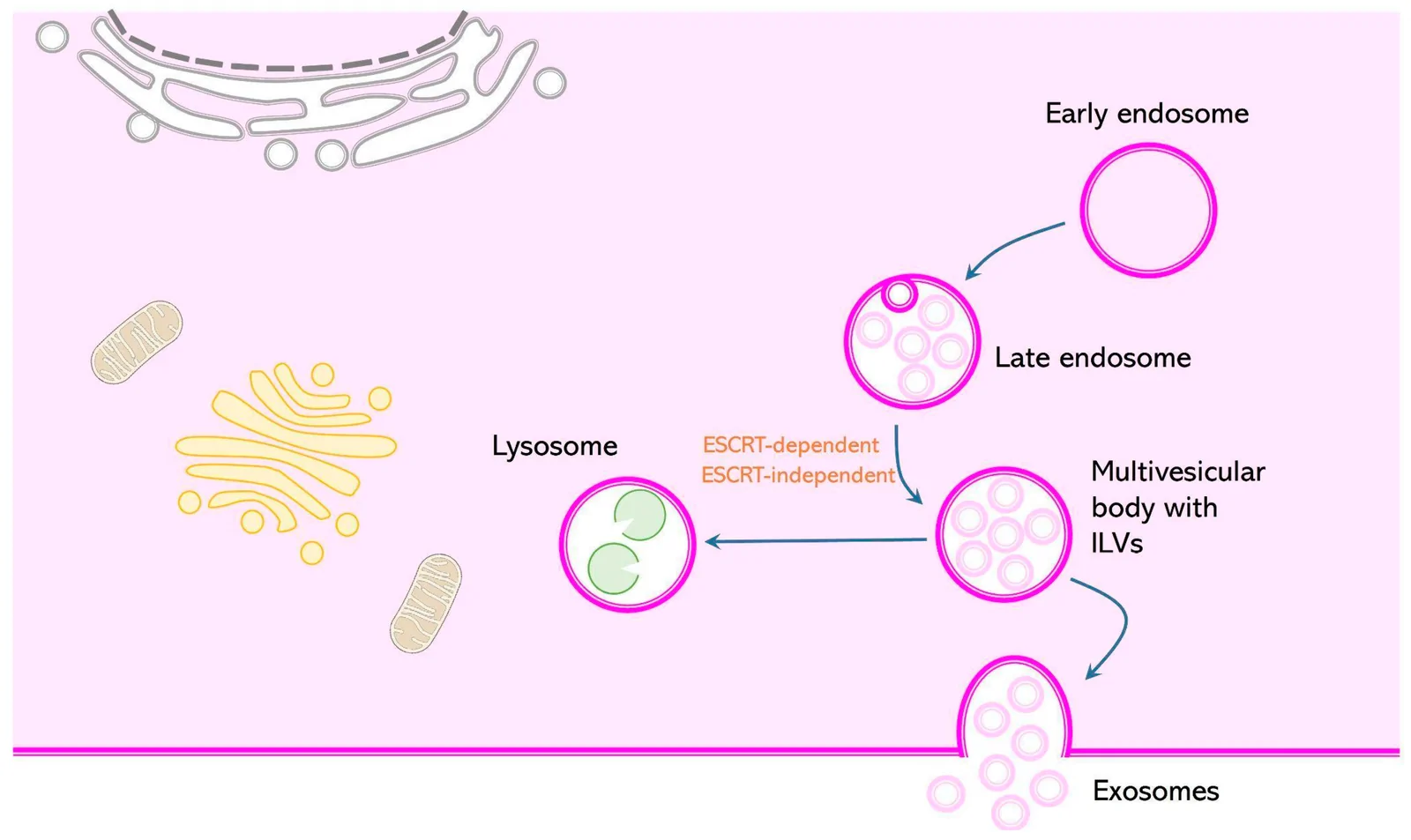
Schematic representation of exosome biogenesis. This process starts with plasma membrane invagination, producing early endosomes that mature into late endosomes. Further membrane invagination generates multivesicular bodies (MVBs) filled with intraluminal vesicles (ILVs), a process regulated by ESCRT-dependent or independent pathways. MVBs are subsequently trafficked for either lysosomal degradation or fusion with the plasma membrane. Exocytosis of MVBs releases ILVs into the extracellular space, where they are termed exosomes.

Exosomes are biological nanoparticles within the group of extracellular vesicles, which are crucial mediators of information transfer between cells under normal and diseased conditions [8]. There are three main groups of EVs that differ in size, biogenesis mechanisms, and properties: microvesicles (MVs), apoptotic bodies, and exosomes [8,9]. MVs (100–1000 nm) are generated by budding and shedding from the plasma membrane, while apoptotic bodies (>1000 nm) are released by cells destined for programmed cell death [8,9]. Exosomes are generated as intraluminal vesicles (ILVs) via the endosomal pathway [8,9]. This biogenesis process begins with the invagination of the plasma membrane, leading to the formation of early endosomes [8]. Subsequently, the endosomal membrane invaginates (late endosome), generating multivesicular bodies (MVBs) with intraluminal vesicles [8]. During this process, cytosolic components, including proteins, lipids, and RNAs, become encapsulated within the ILVs [8,9]. The MVBs eventually fuse with the plasma membrane, releasing the ILVs into the extracellular milieu as exosomes (Figure 1) [8,9].

The biogenesis process is strictly regulated by two pathways: the endosomal sorting complexes required for transport (ESCRT)-dependent pathway and the ESCRT-independent pathway [7,8]. In the ESCRT-dependent pathway, the ESCRT machinery facilitates vesicle budding and cargo sorting [8]. The ILV sorting process is primarily mediated by the recognition of ubiquitinated proteins and, to a lesser extent, by the recognition of G protein-coupled receptors mediated by ALIX, an ESCRT accessory protein [8,9]. ALIX also interacts with syntenin-1 and syndecan to form the Syndecan–Syntenin–ALIX complex, modulating exosome biogenesis [8]. Within the ESCRT-independent pathway, the ceramide pathway is distinguished, which is based on sorting according to the lipid composition of the endosomal membrane, where high concentrations of sphingolipids form microdomains [8]. In addition, the tetraspanin pathway is also present, with membrane proteins that interact with different transmembrane receptors to form tetraspanin-enriched microdomains, which serve as cargo transport platforms [8,9]. Exosomes are enriched in the tetraspanins CD9, CD63, and CD81, so they are often used as biomarkers of this type of extracellular vesicles [8,9].

Following MVB formation, they can be degraded by fusing with lysosomes or secreted into the extracellular space, releasing exosomes [7,8,9]. The transport and docking of MVBs to the plasma membrane, and the exosome release, require several key molecules, particularly members of the Rab GTPases for vesicular trafficking and the soluble N-ethylmaleimide-sensitive factor attachment protein receptors (SNAREs) for membrane fusion [7,8].

#### 1.2.2. Molecular Composition and Functions

**Figure 2:**
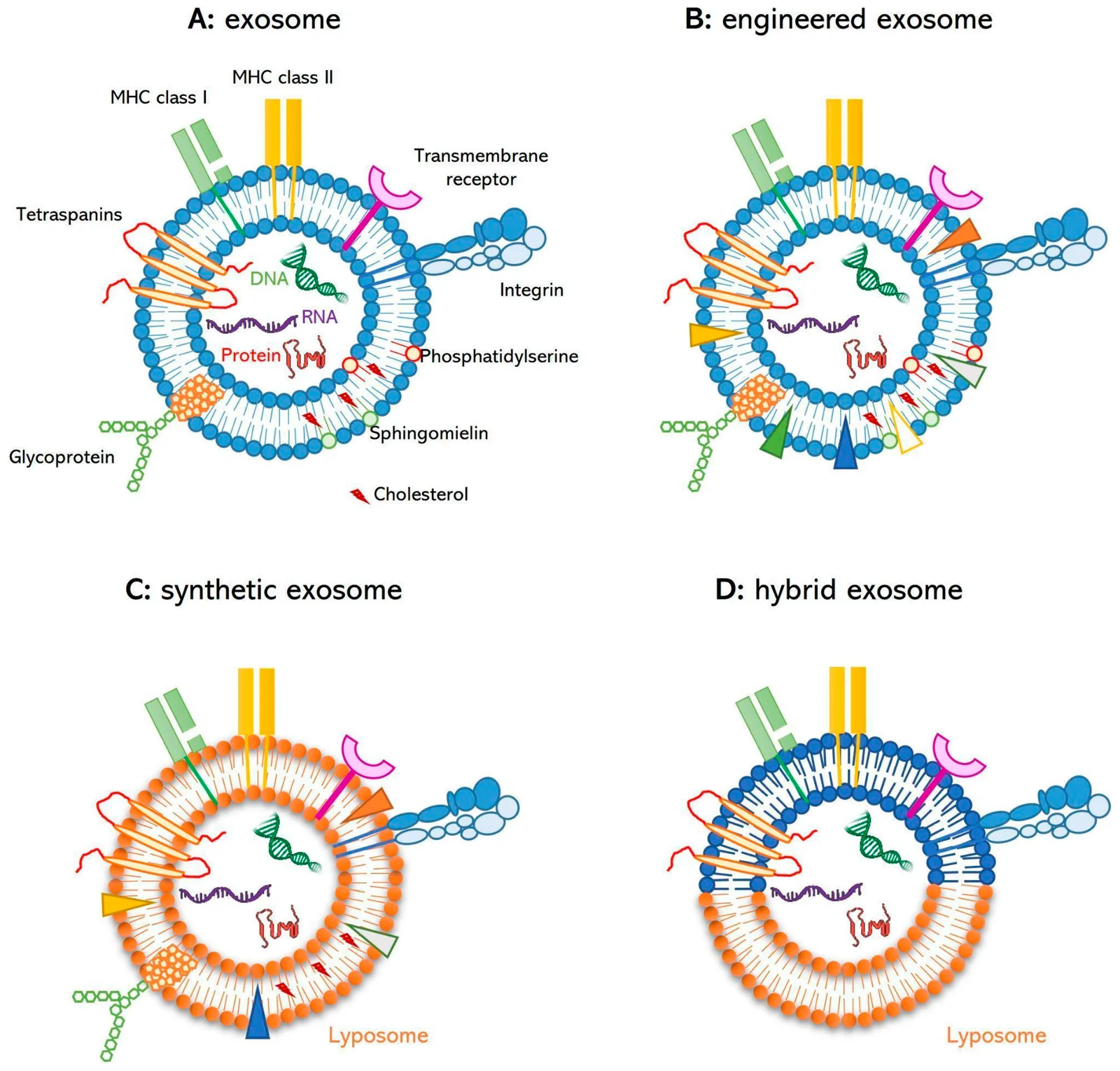
Schematic representation of an exosome and the three types of biomimetic exosomes. (**A**) Natural exosome with different molecules attached to its membrane and a cargo inside. (**B**) Engineered exosome, with its surface modified using genetic or chemical approaches. (**C**) Synthetic exosome, a liposome-based nanovesicle with exosome features and modifications on its surface. (**D**) Hybrid exosome, a combination of an exosome with a liposome. Triangles represent modifications performed on the ELNVs’ surface.

Exosomes are formed by a membrane that is a lipid bilayer, and an interior that contains different types of molecules (exosome cargo) [7,8,9,10]. The transported content or cargo of exosomes consists of nucleic acids, proteins and lipids (Figure 2A) [7,8,9]. Regarding proteins, exosomes can contain those involved in their own biogenesis (ALIX and Tsg101), tetraspanins (CD9, CD63, CD81 and CD82), heat shock proteins (HSP70, HSC70, HSP90), membrane transport and fusion proteins (Rab GTPases, annexins, flotillin and dynamins), glycoproteins, Major Histocompatibility Complex molecules (MHCI and MHCII) and transmembrane receptors [7,8,9,10]. Additionally, lipids that can be present in exosomes include cholesterol, phosphatidylserine, sphingomyelin, ceramides, fatty acids, and phospholipids, which are especially important for cell structure, biogenesis, sorting, and signaling. Finally, nucleic acids are also present in exosomes, including RNA (mRNA, microRNA (miRNA), long non-coding RNA (lncRNA) and DNA [8,9,10]. These nucleic acids play important roles in cellular communication, biological responses, and disease pathogenesis, with miRNAs being particularly abundant and studied as potential non-invasive biomarkers [7,8,9,10]. Overall, exosomes have been shown to contain up to 9769 proteins, 3408 mRNAs, 2838 miRNAs, and 1116 lipids [8,9,10].

The heterogeneity of exosomes likely reflects their size, content, functional impact on recipient cells, and cellular origin. This size inequality could be due to either irregular invagination of the limiting membrane of the MVB, resulting in distinct total fluid and solid contents, or to isolation methods that include other EVs. Refined EV fractionation methods have revealed that exosomes can contain subpopulations defined by distinct size ranges [11]. Size heterogeneity can also lead to different amounts of exosomal content. The organ and tissue of origin of exosomes, along with the cellular microenvironment and intrinsic cellular biology, can also influence their content and biological markers. Similarly, not all exosomes contain comparable amounts of a given cargo, as has been observed, for example, with exosomal miRNA cargo. The combination of all these features could lead to increased complexity and heterogeneity of these NPs [11]. Therefore, it is important to highlight that the effects of exosomes on recipient cells can be different due to their varied expression of cell surface receptors; this functional heterogeneity can give rise to exosome assemblies that induce cell survival, apoptosis, immunomodulation, etc., in different target cell types [11].

Exosomes perform important biological functions, being a protein quality control mechanism for cells [10]. This is because they allow cells to rapidly and selectively remove proteins from the plasma membrane. Examples include the removal of the transferrin receptor from maturing reticulocytes and the expulsion of the sperm receptor Juno from the plasma membrane of mammalian zygotes after fertilization [10]. They are also involved in the vesicular secretion of the cell death-mediating protein MLKL, the expulsion of unarmored and chemically modified RNAs, and the secretion of neurodegenerative amyloidogenic proteins and aggregated signaling molecules. Moreover, their biogenesis contributes to anterior–posterior cell polarity, key for migratory cells (i.e. leukocytes), by establishing a polarized protein sorting pathway [10].

Exosomes also act as components and remodelers of the extracellular matrix and contain tissue factors, so they can serve as templates for thrombus initiation in wound repair and in pathological conditions such as paraneoplastic coagulation [10,11]. Similarly, they are platforms for producing diffusible signaling molecules such as adenosine, prostaglandins, and lysophospholipids, and contribute to the formation and propagation of amyloid-rich aggregates in neurodegenerative disorders [10]. Furthermore, a key function of exosomes is their involvement in intercellular signaling, as they can transmit complex signals by interacting with cell surface receptors. Notably, they also participate in molecular transfer, as exosome fusion with target cells can deliver functional receptors and even activated receptors and effectors to those cells. Their importance has been demonstrated in PD-L1 signaling in cancer immunosuppression, microRNA reprogramming during pregnancy and viral infections, and EGFRvIII transfer in cancer. Viruses, such as HIV, can also utilize exosome biogenesis pathways for the assembly of infectious particles and the establishment of host permissiveness [10,11]. As a result of these properties, exosomes are being developed as therapeutic agents in various disease models [10].

### 1.3. Biomimetic Exosomes

Exosome-like nanovesicles (ELNVs), also known as biomimetic exosomes, are an innovative approach that has arisen to overcome the drawbacks of naturally occurring exosomes, such as difficulty in isolation, cargo loading, stability, and production [12,13,14]. NPs have a highly stable structure, are homogeneous, and can be easily surface-modified. Nanoparticle biocompatibility depends on size, shape, charge, and chemical composition [15]. While organic NPs are highly biodegradable and well-tolerated, inorganic NPs pose long-term toxicity risks due to organ accumulation [16]. To improve their safety and biological behavior, surface engineering is used. Coating NPs with polyethylene glycol (PEG) is the gold standard; it forms a hydrophilic shield that blocks protein adsorption, evades immune clearance, and significantly extends blood circulation time [15]. Therefore, the ELNV strategy seeks to combine the strengths of both worlds to improve large-scale production and cell targeting, among others [13,17,18].

There are three different kinds of ELNVs: engineered exosomes, synthetic exosomes and hybrid exosomes (Figure 2). Engineered exosomes are exosomes whose surfaces have been modified using various methods, including genetic manipulation of the parental cells to increase the expression of a specific protein or ligand, chemical modification, or polymer conjugation to functionalize the exosomal surface [18]. Synthetic exosomes, another kind of ELNVs, are liposome-based nanovesicles with exosome-like features modified with peptides to enhance their cell-targeting capacity, while overcoming the low-yield limitations of biologically derived exosomes [13,18]. Finally, hybrid exosomes are nanovesicles created by the fusion of exosomes with liposomes using different techniques. The goal is to obtain benefits from the synthetic vector (the liposome) and the natural exosome properties [12,17].

### 1.4. Applications in Biomedicine

The biomedical applications of natural and biomimetic exosomes depend on both their molecular composition and their ability to be taken up by specific recipient cells [4,11,19]. Four main mechanisms for their internalization have been proposed: endocytosis, phagocytosis or macropinocytosis, ligand–receptor interaction, and direct fusion [4,11,19]. Notably, endocytosis appears to be the main method of entry of extracellular vesicles, especially clathrin-mediated endocytosis, since it is one of the canonical pathways for exosome uptake [11,19]. Previous studies demonstrate that endocytosis is usually a rapid process influenced by temperature, and that exosomes are internalized in recipient cells 15 min after initial contact [19]. Another mechanism of intercellular communication mediated by exosomes consists of the interaction of exosomal membrane proteins with receptors present on target cells, which activate the corresponding intracellular signaling [4,11,19]. Several classic ligand/receptor pairs have been described in these interactions, each of which is likely specific to a particular exosomal cellular source and recipient cell type [4,11,19]. Finally, direct fusion between the plasma membrane of a recipient cell and the subsequent transfer of its contents, favored by an acidic pH, possibly due to differences in lipid content or the overall ionic charge of the exosome surface after its release, is an important mechanism in exosome internalization [11,19].

Although extracellular vesicles can be internalized by recipient cells and, in some cases, cross biological barriers, their uptake does not necessarily imply membrane crossing or cytosolic cargo delivery [20,21]. Endosomal trafficking, degradation, recycling, and membrane fusion can determine the ultimate fate of the vesicle and its cargo [22].

The uptake of exosomes is of great importance for their application in biomedicine in diagnostics, gene and regenerative therapy, drug delivery, and immunotherapy [4,11,19,20]. In terms of diagnostics, natural exosomes are found in all biological fluids and are released by all cells, making them an ideal tool for minimally invasive liquid biopsies that allow monitoring disease progression [4,11,19,20]. Their complex molecular cargo offers a multiparametric diagnostic window, and their surface proteins facilitate their detection [11,19,20]. They are being applied in the diagnosis of cardiovascular diseases, central nervous system diseases, and, especially, cancer [4,11,19,20]. As biomarkers, exosomes can contain DNA with cancer-associated mutations, such as KRAS and TP53 [11,20]. They are also a rich source of specific miRNAs, whose differential expression between cancerous and normal cells can serve for early diagnosis and prognosis in various types of cancer, such as miR-21, miR-155, miR-17-92, and miR-1246 [11,20]. Furthermore, exosomal proteins, such as phosphoproteins and glycan-1, have also proven useful in cancer diagnosis [11,20]. The combination of different molecular payloads (proteins, lipids, RNA, miRNA) seeks to improve diagnostic specificity and sensitivity [11,20].

Similarly, these small extracellular vesicles hold promise as therapeutic agents on their own or as drug delivery vehicles, as they appear to undergo efficient cellular internalization and exhibit low immunogenicity [4,7,11,19,20]. However, the immunogenicity of EVs—including exosomes (30–200 nm)—is a complex process governed by cellular origin, structural properties, and processing methodologies [23]. Cellular origin dictates EV immunogenicity: MSC-derived EVs are immunosuppressive with low intrinsic immunogenicity, whereas allogeneic or xenogeneic EVs trigger rapid adaptive and innate clearance [23]. Mesenchymal stem cell-derived exosomes have been shown to be well tolerated in patients with graft-versus-host disease [11]. Surface composition further regulates immunogenicity: CD47/SIRPα signaling inhibits phagocytosis, PD-L1 drives immune tolerance, and B2M knockout reduces MHC-I levels to minimize adaptive immunity [23]. Exosome surface engineering with CD47 triggers the SIRPα “don’t eat me” signal on phagocytes, suppressing immune clearance [23,24]. This strategy creates targeted immunoprotection without systemic toxicity, reducing rejection in cell therapies and xenotransplants while explaining tumor immune evasion [24].

Moreover, exosome targeting is an important issue in drug delivery. Natural EV cell tropism and biodistribution are governed by surface integrins, tetraspanins (e.g., Tspan8, CD63), membrane lipids (e.g., phosphatidylserine), and surface glycans. However, to overcome rapid off-target clearance in the liver and spleen, EVs can be engineered using pre-production genetic edits (e.g., Lamp2b or GPI-nanobody fusions) or post-production surface modifications (e.g., click chemistry, aptamers, or magnetic particles) to achieve precise tissue delivery [25,26]. Therefore, biomimetic exosomes can be designed to target specific tissues by modifying their surface ligands [4,7,11,19,20]. For instance, it has been demonstrated that the use of cartilage-affinity peptides enables exosome targeting to chondrocytes in osteoarthritis models; similarly, aptamers have been shown to direct repair and remyelination in the central nervous system [25].

Regarding the effect of exosomes in drug delivery, they have been loaded with chemotherapeutics such as doxorubicin and paclitaxel to treat cancer, demonstrating antitumor efficacy and reduced toxicity in animal models [7,11,19,20]. Furthermore, they can deliver genetic material, such as miRNAs and siRNAs, protected from degradation, allowing them to act at distant sites [4,7,11,19,20]. This has been tested in cancer, such as the delivery of KrasG12D siRNA for pancreatic cancer (currently in a Phase I clinical trial), and in neurological diseases, where they can cross the blood–brain barrier to deliver siRNA or proteins with neuroprotective effects [7,11,19,20]. Finally, exosomes are also being explored to modulate the immune response, seeking to enhance antitumor responses [4,11,19,20]. For example, dexosomes (derived from dendritic cells) have been shown to increase the cytolytic activity of NK cells in patients with lung cancer [4,11,19,20].

Table 1, Table 2 and Table 3 provide several examples of therapeutic applications of exosomes and biomimetic exosomes as drug delivery vehicles for the treatment of cancers, in regenerative medicine, and in ocular therapies. The concepts listed in these tables are described in detail in the following sections. It should be noted that, in the exosome studies, the source of these nanoparticles, the isolation or synthesis method, the characterisation methods, the load and the encapsulation method can be very diverse. Within this variety, both natural and biomimetic exosomes have great therapeutic potential, although the target specificity is generally increased in the latter.

## 2. Exosome Isolation Methods

The development of efficient and standardized isolation methods that allow obtaining pure and homogeneous exosomal preparations in sufficient quantities is a key requirement for the translation of exosomes to clinical practice [42]. The most widely used approaches currently rely on their known physical (size, shape, density, charge) or chemical (membrane surface composition) properties [43]. These characteristics enable EV enrichment (single step), separation from other components (at least two consecutive steps), or subpopulation fractionation [44]. Recently, multiple studies have evaluated combined isolation methods—leveraging orthogonal or complementary principles—to improve EV specificity across various biological fluids and to increase the purity of the sample [44]. However, the high complexity of these fluids and the lack of specific exosomal markers with high expression levels make obtaining pure exosome fractions without losses extremely difficult [43].

In addition to exosomes, other components of similar size, mass, and density circulate in any biological fluid: proteins, lipoproteins of different densities, nucleoprotein complexes, other types of membrane vesicles, etc. [42,43]. These biologically active particles, like exosomes, can contain miRNAs or signaling proteins and lipids. Another inherent problem with their isolation is that the exosomal reservoir itself is quite heterogeneous, containing subpopulations of nanovesicles that differ in size, morphology, surface markers, and biochemical content [42,43].

Moreover, the nature of the starting material has a major impact on the selected exosome isolation methods and their efficacy [43,45]. The most used sources for their isolation include cell culture media, plasma, serum, urine, saliva, cerebrospinal fluid, and milk [45]. Plasma is a complex biological fluid containing exosomes along with cell debris, apoptotic bodies, microvesicles, and plasma proteins [43,45]. This complicates exosome isolation based on size and biochemical properties, as these characteristics are similar across components [43]. Urine and cerebrospinal fluid have fewer interfering particles than blood or plasma but a lower concentration of exosomes, and therefore require larger volumes for equivalent amounts [43,45]. Regarding cell culture media obtained after cell cultivation, they are widely used for mass production because they are simple, inexpensive and do not require animals or human subjects [45]. Cell culture media may have a higher yield of exosomes than serum or plasma [43,45].

**Figure 3:**
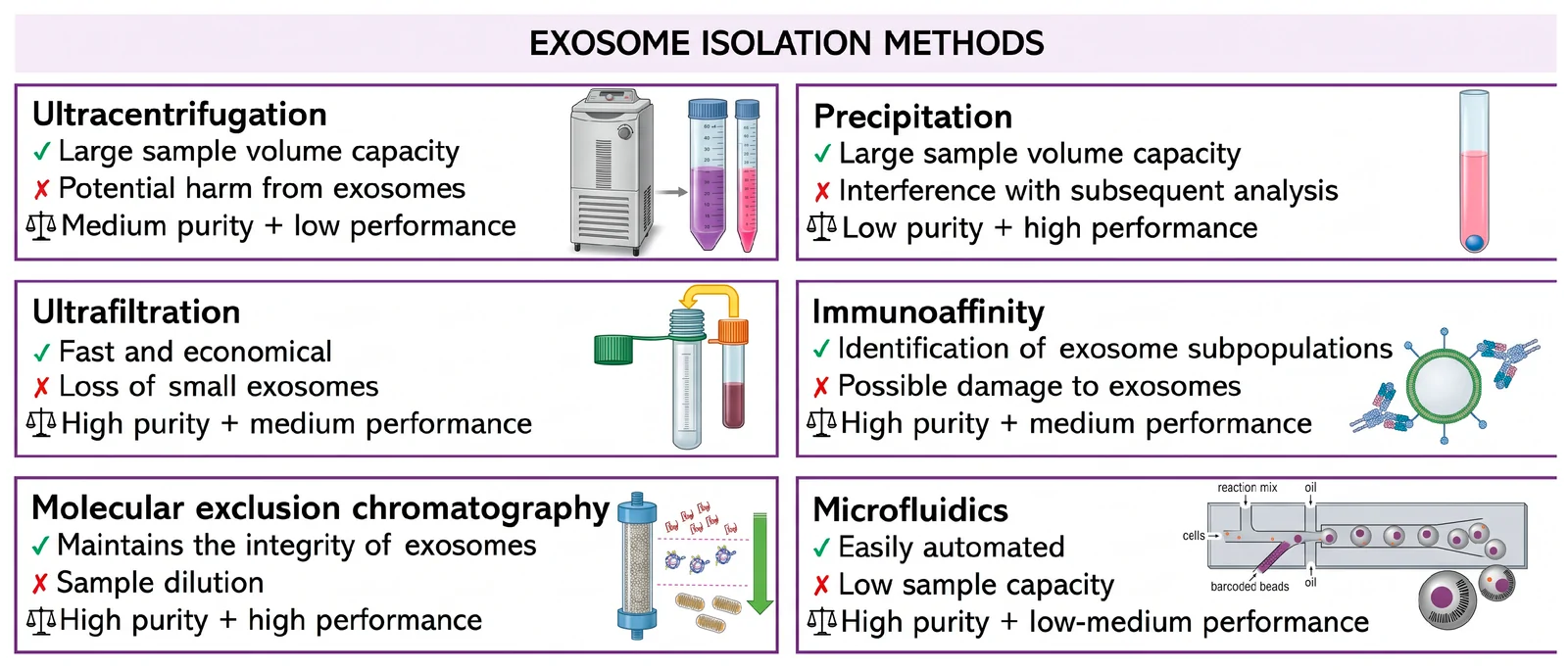
Diagram of the most common methods for isolating exosomes. Green tick symbols (✔) indicate the advantages of the methods, while red crosses (✘) refer to the disadvantages. In all cases, the level of purity and yield of the technique is indicated.

This section presents the most widely used techniques for isolating exosomes (Figure 3) [8,43,46,47,48]. The aim of these is to obtain the purest possible populations of exosomes with high yields [42,43].

### 2.1. Separation Based on Density

Ultracentrifugation is considered the gold standard for EV (including exosomes) isolation and separates molecules based on their shape, size, mass, and density by subjecting them to very high centrifugal forces (up to 1,000,000 *g*) [46]. Differential ultracentrifugation and density gradient ultracentrifugation are the two most popular types of preparative ultracentrifugation [8,46]. However, as with other non-specific isolation techniques—such as size-exclusion chromatography, filtration, and precipitation—it is necessary to confirm the presence of exosomes through molecular marker analyses (e.g., presence of CD63, CD9, CD81, Alix, and TSG101, and absence of calnexin) or physical and structural characterization, as described in Section 4 [44,49,50].

#### 2.1.1. Differential Ultracentrifugation

The procedure followed in this technique is carried out at 4 °C and begins with low-speed centrifugation cycles (300–400 *g*) to remove cells [43]. Speed is then increased to 1000–2000 *g* to remove cell debris and apoptotic bodies [8,42]. The EVs are then centrifuged at 10,000 *g* to remove microvesicles. Finally, the EVs are precipitated by high-speed centrifugation, typically between 100,000 and 200,000 *g* [43]. The EVs pellet can be resuspended and centrifuged again to increase purity, although this might reduce the yield [8,42,45].

The advantages of this technique are its low cost, the absence of sample pretreatment, the ability to process large sample volumes (more than 400 mL), a high exosome yield, ease of use, and minimal technical expertise [8,42]. However, it has certain drawbacks: it is laborious and time-consuming, has low reproducibility, and can present low purity due to protein impurities and other aggregates of EVs, virions, and other molecules [8,42,43,45]. Furthermore, multiple high-speed centrifugation cycles can damage the structure and integrity of the isolated exosomes [42].

#### 2.1.2. Density Gradient Ultracentrifugation

Density gradient ultracentrifugation is a modification of differential ultracentrifugation that aims to improve the purity of isolated exosomes. In this case, particles are separated based on their size, mass, and density using a density gradient medium, such that the particles are concentrated in the gradient region corresponding to their buoyant density [8,42,43,45]. The procedure followed in this technique consists of subjecting the samples to low-speed centrifugation to remove cells and larger debris. They are then loaded into a density gradient separation solution and centrifuged (e.g., 100,000 *g* for 16 h) [42,43]. During this time, the exosomes are concentrated in the corresponding density gradient region (1.13–1.19 g/mL) to remove other contaminants [8]. Sucrose or iodixanol is commonly used to form the density gradients [45].

There are two variants: isopycnic and moving zone [8,42,43]. In the first, the density gradient of the separation medium corresponds to the densities of all the components of the sample. The particles move through the gradient and accumulate in the region that coincides with their buoyant density [42]. In this method, the fractions remain in that region throughout the centrifugation cycle, without sedimenting to the bottom with longer centrifugation times [8,42]. In the second, the particles are separated according to their size and mass. The sample is loaded as a thin layer on the top of a pre-made density gradient medium that is characterized by having a lower density than all the particles in the sample. The particles move along the gradient and are separated according to their size and mass, not just by density. A high-density “cushion” is often used at the bottom of the centrifugation tube to prevent exosomes from completely settling to the bottom [42,43]. This is important because, unlike isopycnic ultracentrifugation, where particles are stopped at their buoyant density, in velocity zonal ultracentrifugation, all particles will precipitate to the bottom if centrifugation time is too high [8,42,43]. Therefore, this variable must be optimized to prevent exosomes from completely settling [8].

Density gradient ultracentrifugation has advantages such as a significant improvement in the purity of isolated exosomes compared to differential ultracentrifugation, as well as the possibility of separating exosome subpopulations [43]. However, this technique considerably increases the processing time (more than 16 h), requires specialized labor, specific expensive equipment, and the processing volume is limited by the thin loading zone [42,43]. Furthermore, isopycnic density gradient ultracentrifugation cannot efficiently separate vesicles with similar buoyant densities (such as microvesicles), even if they differ in size [43].

### 2.2. Separation Based on Size

Ultrafiltration, tangential-flow filtration and size-exclusion chromatography (SEC) are based on the separation of these extracellular vesicles based on the size difference between these NPs and the components of biological samples [51].

#### 2.2.1. Ultrafiltration

Ultrafiltration uses membranes with defined pores (10–100 nm) to isolate EVs from the rest of the components present in a biological sample (plasma, serum, urine, or cell culture medium) [8]. Pressure is applied to pass the liquid sample directly through the membrane pores (traditional dead-end filtration), allowing the separation of particles according to their size [43,45]. By adjusting the filter pore size, it is possible to classify specific subpopulations of small extracellular vesicles, including exosomes, according to their defined size [46]. Notably, several ultrafiltration configurations are used: tandem ultrafiltration and sequential ultrafiltration [8,43,45,46].

In tandem ultrafiltration, two filters are connected in series with size exclusion limits typically ranging from 20 to 200 nm [8,43]. Large particles (cell debris, apoptotic bodies, and microvesicles) are trapped by the first membrane, while smaller particles (proteins) pass through the second filter. Exosomes (30–150 nm) and other NPs with similar size are trapped by the middle membrane or the 20 nm filter. In contrast, in sequential ultrafiltration, the biological fluid is first filtered through a 1000 nm filter to remove large particles, and the resulting filtrate then passes through a second filter with a 500 kDa molecular weight cut-off that removes free proteins and other smaller particles. Finally, EVs, including exosomes, are collected from the filtrate using a 200 nm filter. In addition to the above-mentioned ultrafiltration configurations, centrifugal ultrafiltration and tangential flow filtration can also be performed [8,43,45,46].

Ultrafiltration as an exosome isolation method offers certain advantages, such as its low cost, simplicity, speed, high efficiency, and wide range of sample volumes, allowing both the processing of small quantities and large-scale exosome isolation [43,45]. However, this method offers moderate purity and yield due to potential obstruction of membrane pores and vesicle entrapment. Furthermore, the pressure applied during the filtration process can generate shear stress and damage the structure and integrity of exosomes, affecting their biological functionality and, consequently, their subsequent therapeutic applications [43]. Similarly, contamination in the isolated sample can occur due to the presence of nanoparticles with sizes comparable to those of exosomes [45]. Therefore, one possible solution is to combine ultrafiltration with other techniques such as centrifugation or size exclusion chromatography to improve the purity and yield of isolated exosomes. These combinations seek to profit from the advantages of each method and compensate for its limitations [43,45].

#### 2.2.2. Tangential Flow Filtration

Unlike traditional dead-end filtration, tangential flow filtration (TFF) directs fluid parallel to the membrane surface rather than perpendicularly. This configuration minimizes membrane fouling, enabling the efficient concentration and filtration of liquid samples using high molecular weight cut-off membranes, usually between 100 and 500 kDa [52,53].

The design of TFF systems is relatively straightforward and obviates the need for highly specialized equipment, making the technique particularly cost-effective when membranes are regenerated. Since pore sizes can be precisely adjusted to suit specific applications, TFF has proven highly versatile for the bench-scale isolation of numerous bioparticles, ranging from EVs and viruses to plasmid DNA nanoparticles [54]. Moreover, TFF is beneficial compared to centrifugation approaches as the EVs have undergone no centrifugal force, promoting the recovery of intact EVs with little structural damage. However, the choice of MWCO membrane, pressure, and flow rate are critical variables in ensuring intact EVs with high purity [53].

TFF also holds significant translational potential, especially when combined with size-exclusion chromatography. Multiple studies have demonstrated that TFF-SEC outperforms conventional methods by providing superior separation efficiency, higher yields, and enhanced purity. Consequently, this approach is highly suitable for large-scale processes that demand high recovery rates [43,55,56]. Moreover, TFF significantly streamlines drug commercialization by maintaining a single process design throughout development. Because material volume scales proportionally to membrane surface area, one core TFF process scales effortlessly from the bench to pilot studies and commercial manufacturing [54].

#### 2.2.3. Size Exclusion Chromatography

Gel permeation chromatography is a technique used for exosome isolation based on differences in the hydrodynamic or Stokes radii of EVs [8]. As a reference method for separation and purification, it can be seamlessly incorporated into integrated production workflows. This technique separates biomolecules based on their hydrodynamic size as they pass through an inert, low-adsorption resin, consisting of a porous matrix of beads packed in a column [8,43]. The molecules are entrained by a mobile phase, so that the largest particles elute first, not penetrating the pores [43,57]. In contrast, smaller particles and molecules penetrate the pores to varying degrees depending on their size, so their elution time increases as their size decreases [8,43,57].

The main advantage of gel-exclusion chromatography is that it preserves exosome integrity and biological activity, unlike other techniques such as ultracentrifugation and ultrafiltration, which apply high forces and pressure [43,46,57,58]. This type of chromatography is performed using passive gravity flow, which minimizes damage to the exosome structure and therefore facilitates functional studies and subsequent therapeutic applications [43,46,58]. Furthermore, it is a reproducible technique applicable to different types of samples, allowing one to produce exosomes with high purity since protein and lipoprotein aggregates are efficiently separated [43,46,57,58]. Similarly, the required sample volume is minimal, with possible volumes as small as 15 µL with commercially available columns to achieve high resolution [43,58]. Furthermore, it is a rapid method, does not require complex sample pretreatment, and is easily scalable and automated for high-throughput exosome preparation, with systems available on the market [43,57,58]. Additionally, combining this process with tangential flow filtration maximizes final sample purity, as mentioned above. Despite the aforementioned advantages, gel-exclusion chromatography also has certain drawbacks, such as sample dilution, which could require additional steps to concentrate the exosomes [43,57,58].

### 2.3. Separation by Precipitation

Exosome separation by precipitation is an isolation technique based on altering the solubility of particles in solution using hydrophilic polymers [8,43,45,59]. This method involves the addition of hydrophilic polymers such as polyethylene glycol (PEG) to the sample containing exosomes [8,43,45,59]. PEG, being highly hydrophilic, interacts with the water molecules in the sample, creating a hydrophobic microenvironment that reduces the solubility of the exosomes and causes their aggregation and subsequent precipitation [8,43,59]. These EV aggregates can be collected by low-speed centrifugation (1500 *g*) [8,43,59]. The process can form PEG-exosome micelles or microcapsules by self-assembly [59]. The addition of 1 M sodium chloride can help optimize the separation of other small molecules or unwanted proteins by adjusting the ionic strength of the solution and reducing electrostatic interactions between proteins [59].

It should be noted that in precipitation isolation, the biological sample (cell culture media, urine, plasma, serum) must first be pretreated by low-speed centrifugation (300–1000 *g* for 5–10 min) to remove dead cells and cell debris [59]. Additional centrifugation (2000–6800 *g* for 10–45 min) can remove larger vesicles and apoptotic bodies. The supernatant can then be filtered using a 0.22 µm pore filter to remove further non-EV materials [43,59]. The pretreated sample is mixed with an aqueous PEG solution and incubated at 4 °C for more than 12 h to allow aggregate formation [43]. After this time, EV aggregates are collected by low-speed centrifugation (1500–16,000 *g* for 10–60 min) [43,59]. Furthermore, further purification is possible by centrifuging at higher speeds (100,000–130,000 *g* for 30–130 min) [8,43,59].

This precipitation isolation method offers several advantages, such as its ease of use, low equipment requirements, and high yield, as it tends to produce a high quantity of exosomes, often between 5 and 14 times greater than those obtained by ultracentrifugation [8,43,45,59]. This makes it an easily scalable method for processing large volumes of samples, which is advantageous for clinical applications [8,43,45,59]. It also preserves the integrity of the exosomes and allows the production of these NPs suitable for subsequent DNA, RNA, and protein analysis [8,45,59]. However, this method presents a low purity of the final product, as PEG can coprecipitate various impurities, including lipoprotein aggregates, viruses, and the residual polymer itself [8,43,45,59]. This contamination could affect subsequent analyses and falsify exosome quantification, as it is often based on total protein content [8,43,59]. Furthermore, it is a nonspecific technique: it cannot differentiate exosomes from other particles of similar size and molecular weight [43,45,59].

Finally, there are several commercial kits based on the principle of PEG precipitation from plasma and cell cultures, such as ExoPrep (HansaBioMed, Tallinn, Estonia), Total Exosome Isolation Reagent (Invitrogen, Thermo Fisher Scientific, Waltham, MA, USA), ExoQuick (System Biosciences, Palo Alto, CA, USA), and miRCURY Exosome Isolation Kit (Exiqon, Vedbaek, Denmark) [59]. Although all of these are suitable, the purity and yield of the isolated exosomes may vary depending on the kit [59,60].

### 2.4. Immunoaffinity Separation

Exosome isolation by immunoaffinity capture is a highly specific separation technique based on the selective interaction between protein markers present on their surface (CD9, CD63, CD81, ALIX, EpCAM, RAB5 and ANNEXIN) and immobilized antibodies [47]. These membrane peptides establish a precise union with the corresponding antibodies, which are fixed to a solid substrate, so that this matrix is enriched with exosomes [43,47]. For their isolation, a final elution step is required to separate these extracellular vesicles from the solid surface [43].

Within this class of separation, three types are distinguished depending on the variation in the coated antibody substrates: stationary-phase chromatographic separation, immunoaffinity separation with magnetic beads, and enzyme-linked immunosorbent assay (ELISA) [8,47]. In enzyme-linked immunosorbent assay (ELISA), antibodies are immobilized directly onto the surfaces of microplates [8,47]. This is a common method for isolating and quantifying exosomes since it allows them to be captured and quantified, for example, using an anti-CD9 antibody-based system to analyze urine and blood samples [47]. In contrast, in immunoaffinity separation with magnetic beads, the beads are coated with specific antibodies (e.g., anti-CD81, anti-CD63, anti-CD9) to capture exosomes expressing these markers [8,47]. Its use has been demonstrated for isolating exosomes from melanoma using an anti-CSPG4 monoclonal antibody with an efficiency of 98% [47]. Magnetic nanoparticles combined with transferrin have also been used to isolate exosomes derived from brain blood in neurological diseases [47].

In general, immunoaffinity separation offers certain advantages, such as high specificity and purity [8,43,46,47]. It allows for the discrimination of specific subpopulations of exosomes, which is crucial for identifying biomarkers associated with specific diseases [43,46,47]. However, it is important to consider that affinity-based protocols involving the tetraspanins CD9, CD63 and CD81 are not specific for exosomes as an EV subtype; using antibodies to each of these tetraspanins enriches EV populations that do not completely overlap in molecular composition [49]. Furthermore, it also reduces chemical contamination [43,47]. In contrast, it offers a low total yield of exosomes since it only isolates those that express the target markers [43,46,47]. This can introduce analytical bias and underestimate the total exosome population or lead to false negatives, especially in cancer diagnosis, where protein expression can be modulated with disease progression [43,46,47]. Similarly, the antibodies used are expensive, which may limit their large-scale application [8,43,46,47]. Damage to exosomes is also possible, since elution conditions (non-neutral pH or non-physiological buffers) can irreversibly affect their biological function [43,46,47]. Finally, it is worth noting that, to address these limitations, several strategies are being explored, such as the use of aptamers (short DNA or RNA sequences) as alternatives to antibodies due to their lower cost, greater stability, low batch-to-batch variability, ease of scalability, and chemical modification [8,43,47]. Likewise, methods are being investigated to obtain a wider population of exosomes based on the recognition of more universal components of the exosomal membrane, such as saccharide chains (e.g., through lectins) [43,46].

### 2.5. Microfluidics Separation

Microfluidics is a technology that enables the analysis and manipulation of small volumes of fluids using microchannels on a single chip [42,59]. This technique has emerged as a promising tool for exosome isolation and analysis, offering significant advantages over traditional methods [42,59]. Microfluidic devices are designed to exploit differences in the physical and biochemical properties of exosomes, enabling their separation and purification [8,59]. Typical components of these devices include micromixers, valves, microchannels, and pumps [42,59]. There are two main approaches to microfluidic isolation: immunoaffinity-based microfluidics and physical property-based microfluidics [8,42,59]. The former relies on the specific binding of protein markers on the surface of exosomes (such as CD9, CD63, or CD81) with antibodies immobilized on the microchip [8,19,42,59]. The latter, in contrast, separates these extracellular vesicles based on differences in size, density, and molecular weight between exosomes and other particles [8,42,59]. Therefore, microfluidics offers several important benefits, including miniaturization, low sample and time consumption, high efficiency and throughput, and potential process automation [8,42,59]. Despite its advantages, microfluidics still faces obstacles to widespread clinical adoption due to its lack of scalability (numbering-up needed) and high-cost technology due to chip design and manufacturing [19,42,59].

### 2.6. Comparison Between EVs Isolation Methods

A comparison of the exosome isolation and purification methods discussed above requires evaluating key operational parameters, as summarized in Table 4. Each technique yields distinct recovery rates and purity levels—defined by the presence of protein or lipid contaminants—which directly impact drug delivery potential by affecting exosome integrity. Factoring in process duration, batch-to-batch reproducibility, and overall cost helps define the optimal application for each method. For therapeutic manufacturing, TFF alone and tandem TFF–SEC stand out as optimal integrated production workflows. Finally, rising clinical demand for exosomes has driven next-generation separation platforms—such as EXODUS, magnetic sorting, and affinity capture—that improve isolation speed, purity, and scalability for commercial manufacturing [56].

## 3. Synthesis of Biomimetic Exosomes

The synthesis of biomimetic exosomes depends on the specific type of ELNV and different approaches are employed accordingly [18]. Similar to their natural counterparts, biomimetic variants require post-synthesis purification to remove impurities. The strategies used for this purpose mirror standard extracellular vesicle isolation techniques: while differential and density-gradient centrifugation provide robust EV purification, membrane filtration and size-exclusion chromatography yield narrower size distributions and hold greater translational potential [65].

### 3.1. Engineered Exosomes

This type of biomimetic exosome (Figure 2B) has its surface modified using two main strategies: genetic engineering and chemical modifications. The first strategy consists of attaching the desired ligand, usually proteins and lipids, to exosomal surface transmembrane proteins, e.g., Lamp2b and CD63, or molecules such as glycosyl phosphatidylinositol (GPI). That way, via transfecting host cells with a plasmid containing these fused proteins, modified exosomes with engineered peptides on their surface. This technique promotes the delivery of the exosome to its final target cell and enhances its therapeutic efficacy. However, it also presents limitations: it is not suitable for non-genetically encodable motifs, it requires plasmid generation, and achieving high levels of protein expression in host cells can be complex [12,66,67].

Chemical modification performed on exosomes involves attaching functional groups, molecules, or lipid moieties to the native vesicle surface after isolation (or to engineered donor cells prior to release) to confer additional targeting, stability, or therapeutic capabilities while preserving the integrity of the biological membrane. Among the most widely used approaches are covalent surface modifications, with click chemistry, also known as a bioconjugation reaction, standing out as a prominent method that enables a highly specific, bio-orthogonal attachment of diverse cargos. An example is copper-catalyzed azide–alkyne cycloaddition (CuAAC), where exosomal proteins bearing alkyl tags can be selectively coupled to azide-containing reagents; however, this reaction is not site-specific. A different approach is incorporating lipids in the exosomal bilayer, such as PEG-grafted phospholipids (e.g., DSPE-PEG) or cholesterol-conjugated components, to attach functional groups to exosome membranes. The drawbacks of this non-covalent method include potential alterations in the exosome structure or the inherent complexity of the modification process [12,25,66,68].

### 3.2. Synthetic Exosomes

Two main categories exist for generating these artificial nanovesicles (Figure 2C): top-down and bottom-up strategies. Notably, both methodologies enable in situ drug loading integrated with the synthesis process [18].

The bottom-up technique involves assembling the synthetic exosomes from fundamental components and/or decorating their surfaces with functional groups, such as antibodies, proteins, and peptides [55]. The fundamental concept behind this technique is having a defined lipid mixture that mimics the exosome and afterwards modifications are performed on its surface. For the fully synthesized exosomes, a specific lipid mixture is used to assemble the nanovesicles and they are formed in mechanically emulsified water-in-oil (w/o) droplet compartments, with the addition of functional proteins and RNAs to control the cargo content. Whether they are totally artificial or they are liposomes, their surface is functionalized by complex synthetic pathways. With this reproducible strategy, the encapsulation efficiencies are high and the composition of the synthetic exosomes is precisely known and can be optimized; nevertheless, there is a challenge in simultaneously decorating exosomes with multiple functional groups, and difficulties can be found in large-scale production [13,18,25,55].

Top-down strategies are based on the disruption of large cellular components into nanoscale structures to form artificial vesicles. These artificial exosomes retain the biological complexity of natural exosomes, with little heterogeneity [3]. To produce synthetic exosomes using this approach, there are multiple methods: filter extrusion through polycarbonate membranes, filtration, micro-fluidic shearing, sonication, nitrogen cavitation, or chemically induced cell blebbing, all of which generate nanovesicles that retain many membrane proteins of the source cell [18,55]. Some of these approaches are described in greater detail in Section 5 of this review. The advantages of this approach are the potential for scalable generation of biocompatible synthetic exosomes and for an improvement in their practical applicability, whereas the drawbacks are the limited control of the cargo composition and the purification process is more time-consuming [3,18,55].

### 3.3. Hybrid Exosomes

The combination of exosomes and liposomes represents a promising drug-delivery platform (Figure 2D), integrating the endogenous biological functionality of exosomes with the high production yield characteristic of liposomes [18].

Different techniques are available to achieve the fusion between liposomes and exosomes. A simple incubation of both nanovesicles may be sufficient to facilitate fusion, with temperature acting as a critical factor, but the overall efficiency is low [3,12,14,17]. An alternative is using PEG to promote fusion. This strategy is faster than the previous approach, although it remains temperature-dependent and is challenged by the tendency to form aggregates [3]. Alternating multiple freeze–thaw cycles of a liposome–exosome mixture promotes the formation of hybrid exosomes by inducing membrane disruption followed by subsequent fusion. However, this method has not been validated for the incorporation of therapeutic cargo inside [3,12,17]. Co-extrusion of liposomes and exosomes represents another approach to generate hybrids by using a specific pore size and promoting their interaction and fusion through the applied mechanical stress [56,68,69]. This method maximizes hybrid size uniformity but for hydrophilic cargo the percentage of drug encapsulated is low [14]. Another strategy is generating fusion by the combination of microfluidic mixing with sonication, with the maintenance of reproducibility being the primary advantage [12,14,68].

## 4. Analytical Characterization

**Figure 4:**
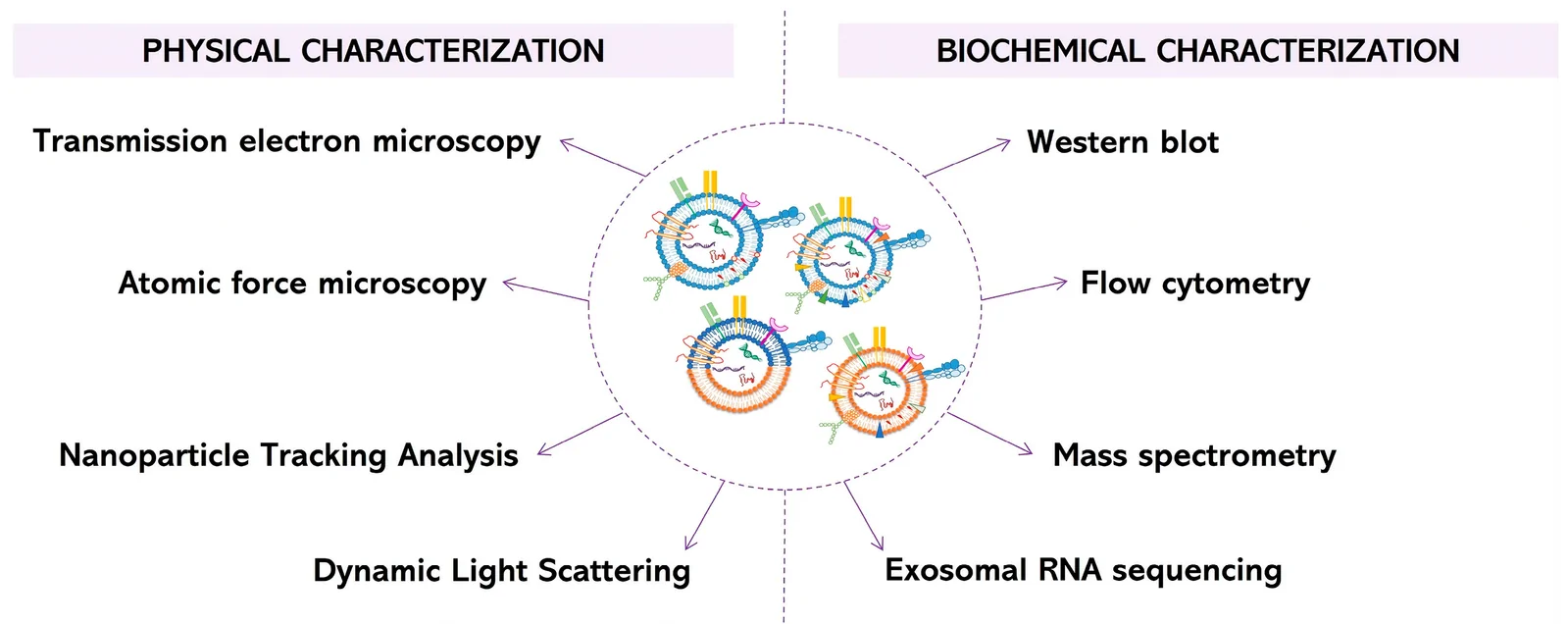
Schematic representation of the analytical characterization workflow for natural and biomimetic exosomes. This characterisation is divided into a physical analysis and a biochemical analysis. The former assesses the size, morphology, charge and concentration of exosomes, using techniques such as transmission electron microscopy (TEM), atomic force microscopy (AFM), nanoparticle tracking analysis (NTA) and dynamic light scattering (DLS). The latter profiled the molecular content of exosomes (RNA, lipids, proteins) employing Western blotting, flow cytometry, mass spectrometry, and exosomal RNA sequencing.

Both the exosomes isolated from body fluids or cell culture media and the biomimetic exosomes synthesized using the methods described above must be quantified and characterized. Two types of analyses are possible: physical characterization and biochemical characterization (Figure 4). Physical characterization studies their size, morphology, surface charge, and concentration, whereas biochemical characterization analyzes their molecular content (proteins, lipids, RNA). Crucially, while the characterization of natural exosomes focuses on deciphering their heterogeneous biological identity and ensuring isolation purity from biofluid contaminants, for artificial variants, it prioritizes validating engineering reproducibility and the fidelity of the biomimetic membrane reconstruction.

This section describes the most widely used techniques for characterizing exosomes, the goal of which is to obtain as much information as possible about them for subsequent applications, especially in the biomedical field [70,71]. As summarized in Table 1, Table 2 and Table 3, researchers employ different combinations of these techniques to characterize EVs, depending on equipment availability and the specific goals of the study.

### 4.1. Physical Characterization

#### 4.1.1. Transmission Electron Microscopy (TEM)

Transmission electron microscopy (TEM) is a widely used technique for characterizing and visualizing biological samples at the nanometer scale, in two dimensions (2D) [71,72]. It is considered one of the most efficient instruments for examining the morphology and size of exosomes, due to its ability to detect particles with a resolution of approximately 1 nm [72]. A transmission electron microscope works under the same basic principles as a light microscope, but uses electrons instead of light [72,73]. In this case, an electron beam passes through a thin layer of the sample, and electromagnetic lenses detect the scattered electrons, creating a diffraction pattern that is captured as an amplified image under vacuum conditions [73].

To analyze exosomes using this technique, the sample is placed on a Formvar carbon-coated TEM grid and incubated for 10 min [71]. Several washes are then performed with distilled water, and the sample is stained using uranyl acetate at room temperature [71]. This staining with heavy atoms with higher electron density is necessary because the biological sample contains elements with low electron numbers (C, H, O). The sample preparation process involves fixation and dehydration [72].

**Figure 5:**
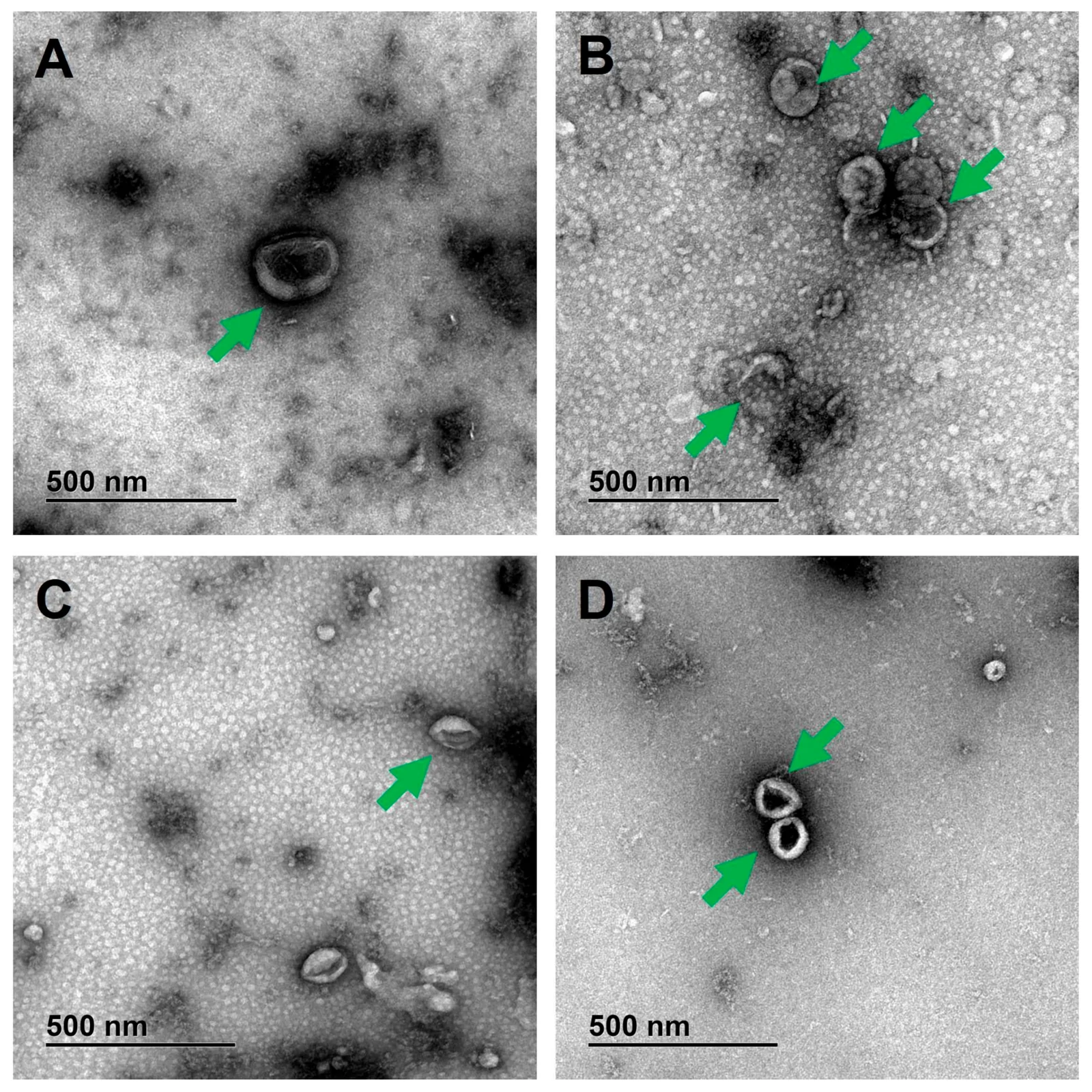
Representative TEM images of exosomes (marked with green arrows) isolated from iPSCs (**A**,**B**) and RPCs (**C**,**D**). Scale bar: 500 nm.

As an example, exosomes have been isolated in our group from the cell culture medium of induced pluripotent stem cells (iPSCs) and retinal progenitor cells (RPCs) obtained by the directed differentiation of iPSCs. For this purpose, an isogenic iPSC line (Oex20.4-B3) generated with the CRISPR/Cas9 system from an iPSC line of an Autosomal Dominant Optic Atrophy plus (ADOA+) patient has been employed [74]. For the isolation of exosomes from the culture medium of iPSCs and RPCs, the Total Exosome Isolation Reagent (Thermo Fisher Scientific, Waltham, MA, USA; 4478359) was used, following the instructions of the manufacturer. Exosomes obtained from iPSCs and RPCs have been characterized using a JEOL TEM-1010 (JEOL, Tokyo, Japan) in ICTS Centro Nacional de Microscopía Electrónica (Madrid, Spain), which operates at 100 kV. Sample preparation was carried out following the aforementioned methodology. Figure 5 shows TEM images with isolated exosomes (marked with green arrows) appearing as cup-shaped structures.

It is worth noting that, for more specific visualization of exosomes, samples can be immunolabeled with primary antibodies that bind to specific exosome proteins and with a secondary antibody bound to a gold nanoparticle [75]. Finally, there is another type of transmission electron microscopy called cryogenic electron microscopy. In this type of microscopy, samples are kept in their natural aqueous environment and do not require dehydration or fixation [71]. Samples are stored in liquid nitrogen (below −175 °C), which helps prevent damage from the electron beam and avoids elemental redistribution or ultrastructural changes. Furthermore, this technique allows for analysis of the interior of EVs, although its equipment is more expensive [71].

#### 4.1.2. Atomic Force Microscopy (AFM)

Atomic force microscopy (AFM) is a technique used to study the surface of a sample with nanometric resolution, without the need for light or electrons [71,76]. It is a type of probing microscopy that uses a very fine (nanometric) tip mounted on a cantilever (flexible lever) [72,77]. This tip travels over the surface of the sample, detecting physical forces between the sample and the tip [71,76]. The interaction forces cause a deflection of the lever, which is recorded by a laser detection system and a position-sensitive detector [72,77]. In this way, with AFM it is possible to obtain high-resolution, three-dimensional (3D) images of the surface topography of a sample that, in the case of exosomes, allows us to determine their size [71,72,77]. AFM is also capable of characterizing the morphology, abundance, biomechanics, and biomolecular composition of exosomes at the nanometric scale [71,76,77].

The AFM technique is characterized by allowing sample measurements under native conditions, with minimal sample preparation and a non-destructive approach [71,72,76,77]. For analysis, exosome samples are deposited on a mica surface and allowed to air-dry at room temperature [71]. After cleaning with ultrapure water, the samples are dried again with nitrogen gas [71]. Alternatively, the mica surface can be coated with antibodies to capture exosomes with specific antigens for visualization, like immunolabeling performed with TEM [71,77]. Despite the topological information provided by this technique with a very small amount of sample, it is an expensive instrument, and sample dehydration can cause topographic modifications [71].

#### 4.1.3. Nanoparticle Tracking Analysis (NTA)

Nanoparticle tracking analysis (NTA) is a widely used biophysical technique for the characterization and quantification of exosomes, allowing the estimation of their concentration and the size distribution of individual particles [71,78,79]. NTA relies on the detection of light scattering by suspended particles and their Brownian motion to estimate the number and volumetric distribution of EVs [71,72,78,79]. NTA can detect and examine vesicles of various shapes and sizes, ranging from 10 to 1000 nm, including exosomes (30–150 nm) [71,78,79]. Concentration data regarding the different extracellular vesicle groups in the sample, including exosomes, can be extracted [71,72,79]. Notably, the NTA system can detect fluorescence, allowing the identification of antigens in exosomes using fluorescently labeled antibodies [71,72,78,79]. This is particularly useful for examining the antigen composition and size distribution in smaller EVs, such as exosomes. Furthermore, this modality allows defining the phenotype of exosome subpopulations and knowing the abundance of each group [71,72].

NTA offers several advantages in quantifying the size and concentration of exosomes in samples isolated from various biological fluids [71,72,78,79]. It is a rapid, efficient, and nondestructive technique that offers high resolution and real-time observation of particle movement [71,72,79]. However, it has some limitations, including the high cost of the equipment and the need for specialized personnel with in-depth knowledge of software and hardware settings to obtain reproducible results [71,78,79]. Furthermore, concentration underestimation can occur, as the sensitivity of the lower limit of detection is low, and exosomes with a smaller size range and a low scattering index may not be detected [72,78]. Another important limitation is the need for adequate sample dilution so that the NTA camera can record all the vesicles present and avoid the masking effect of a larger vesicle on a smaller one [72]. Regarding the fluorescent mode, photobleaching is possible, as well as background interference with the results [72,78,79].

#### 4.1.4. Dynamic Light Scattering (DLS)

Dynamic light scattering (DLS) is a technique that also determines particle size and concentration based on the scattering of light during their Brownian motion [72,79]. However, instead of tracking individual particle motion as in NTA (particle-by-particle analysis), it measures the average fluctuation of scattered light (collective analysis) [73,77,79]. To do this, a coherent, monochromatic laser beam passes through the particle suspension such that the Brownian-moving particles cause constructive and destructive interference, resulting in temporal fluctuations in the intensity of the scattered light [72,77,79,80]. A digital detector monitors these fluctuations to calculate the diffusion rate and size distribution of the particles [72,73,77]. Smaller EVs move faster, causing faster intensity fluctuations and a steeper decline in the correlation curve [73,79]. The particle size distribution is derived from the autocorrelation function of the intensity spectra. Like NTA, the DLS technique calculates particle size using the Stokes-Einstein equation, which relates particle density to its hydrodynamic radius [72,79]. This allows us to obtain the percentage of EVs with a given size, but it cannot calculate their concentration [73,76,77,79].

The DLS technique offers several advantages, such as its simplicity, speed, and good sensitivity for measuring particles across a wide range (1 nm to 6 µm) [72,77,80]. However, DLS does not allow direct visualization of particles and is also unsuitable for polydisperse samples, as larger particles scatter much more light, which can mask the signal from smaller particles and render them undetectable [72,76,77,80]. Signal interference due to aggregates can occur, affecting the accuracy of the analysis [72,80]. Similarly, this technique does not allow determining the absolute concentration of individual particles or of a specific category of particles [73,77,79]. Therefore, although it is a useful tool for obtaining the diameter range of vesicles, it is less frequently used for the direct quantification of exosomes compared to other techniques such as NTA [77,79].

### 4.2. Biochemical Characterization

Biochemical characterization of extracellular vesicles, including exosomes, requires assessing sample presence, purity, and identity via orthogonal approaches, as outlined by MISEV2023 guidelines [49]. To comply, studies must analyze at least one protein marker from each of the primary categories: transmembrane/GPI-anchored proteins (CD9, CD63, CD81), cytosolic membrane-binding proteins (ALIX, TSG101), and non-EV contaminants serving as negative controls. To assess sample purity and rule out cellular debris, these contaminants are evaluated for the absence of co-isolated non-vesicular extracellular particles (NVEPs) [49]. This includes screening for key soluble protein complexes such as apolipoproteins, histones, and calnexin. Optionally, intracellular compartment or protein corona markers may be evaluated to refine EV subtypes. Additionally, non-protein markers (e.g., lipids, nucleic acids) should be validated through co-localization with established protein targets. Moreover, exosomes present different tissue-specific markers that can be analysed, such as L1CAM from the brain or ASGR2 from the liver [81]. Finally, this heterogeneity is also reflected in the pathological and tumor-specific cargo encapsulated by EVs, which allows specific subpopulations to serve as distinct biomarkers for disease diagnosis and monitoring [11,82].

#### 4.2.1. Western Blot

Western blot is a widely used method to detect specific proteins in a sample using antibodies [71,83]. It is based on the separation of proteins by electrophoresis (SDS-PAGE) for subsequent transfer to a membrane (nitrocellulose or PVDF) and incubation with specific antibodies that recognize the proteins of interest; detection is carried out using enzymatic or fluorescent systems [83]. In the case of exosomes, it is possible to use Western blot to characterize them and identify specific proteins of these vesicles, which can be membrane (CD9, CD40, CD63, CD81 and CD151) or cytosolic proteins (Tsg101, ALIX, Hsp70, Hsp90) [71,83,84]. For the artificial exosomes, it is possible to detect specific proteins from the membrane of origin [84].

The electrotransfer technique has advantages, such as its application in the qualitative and quantitative analysis of specifically labeled proteins, allowing both the detection of a specific protein using antibodies and the comparison of expression levels between different groups [71,83]. Furthermore, it is possible to perform early detection of biomarkers associated with certain diseases [71]. However, it is a complex method with a long overall duration (two to three days) and requires a high concentration of proteins for detection [71,83]. Similarly, a specific and universal protein biomarker for exosomes that guarantees that the isolated sample contains exclusively exosomes has not yet been established [71].

#### 4.2.2. Flow Cytometry (FCM)

Flow cytometry (FCM) is an analytical technique that allows the measurement of multiple physical and chemical characteristics of suspended particles one by one as they pass through a laser beam [71,83]. This is because as particles pass through the laser, they scatter light and, depending on whether this scattering is forward or sideways, information is obtained about the size and granularity of the internal structures, respectively [83]. In addition to light scattering, FCM can measure specific molecular components, such as protein or gene expression, or enzymatic activity that have been labeled with fluorescent secondary antibodies [71,83]. Particles can be labeled with fluorochromes inside their interior or immobilized on their surface using monoclonal antibodies. The fluorescence intensity is detected and quantified, allowing the classification of vesicles according to the level of antigen expression [71,83].

Regarding exosome analysis, the sensitivity of conventional flow cytometers (cFCM) is limited to particles larger than 500 nm, leaving these below the detection limit [83,85]. To overcome this limitation, novel and highly sensitive platforms such as high-resolution flow cytometers have been developed, which feature increased sensitivity, enhanced forward scatter detection, fluorescence amplification, and high-resolution imaging to distinguish labeled EVs from background signals [85]. This has enabled the rapid measurement and analysis of exosomes in suspension [83,85]. Furthermore, imaging flow cytometry (IFCM) has been developed, enabling the identification of nanometer-sized particles while maintaining the advantages of cFCM with higher resolution [85]. IFCM has been shown to be a viable approach for exosome analysis, enabling accurate, simple, and high-throughput phenotypic assessment [85]. It also helps clearly distinguish EVs from beads, cell debris, and/or parental cells. Finally, nanoflow cytometry is under development to enable high-throughput analysis of exosomes down to 40 nm [85].

In summary, FCM is a valuable tool for the quantitative and phenotypic characterization of exosomes, especially in clinical samples and for the analysis of specific subpopulations [85]. However, due to its challenges related to the detection of very small particles and the need to differentiate them from other suspended particles, it is often complemented by other techniques for complete and accurate characterization [85].

#### 4.2.3. Mass Spectrometry (MS)

Mass spectrometry (MS) is an analytical technique that identifies and quantifies chemical compounds based on their mass-to-charge ratio [69,83]. It is used for exosome characterization and subsequent protein, lipid, and metabolic profiling, with the study of proteins being the most common of the three [69]. To be analyzed by a mass spectrometer, liquid exosome samples must be ionized [69,83]. The generated ions are separated based on their mass-to-charge ratio using a mass analyzer (quadrupole, ion trap, time-of-flight, or Orbitrap) [69,83]. The amount of ions is measured by a detector, generating a mass spectrum that generally represents a protein fingerprint of the exosome [83]. Unfortunately, numerous components such as SDS, salts, polyethylene glycol, and fetal bovine serum proteins can interfere with MS analysis [83]. Consequently, a highly purified exosome extract is required for accurate and reproducible proteomics [69,83].

There are different MS techniques applied to these EVs, such as liquid chromatography combined with tandem mass spectrometry (LC-MS/MS), which is currently the most widely used methodology to purify exosomes and subsequently characterize their proteins [69]. It allows the simultaneous detection of multiple protein markers due to its high-resolution separation, high throughput, and well-established databases. Nano-LC, and more recently, micro-flow LC-MS/MS, have also been used, offering greater sensitivity and robustness [69]. Likewise, matrix-assisted laser desorption/ionization mass spectrometry (MALDI-MS/MALDI-TOF MS) can be used to analyze intact exosomes without the need for lysis or labeling [83]. These are mixed with a matrix that absorbs laser energy to form co-crystals, allowing for the qualitative and semi-quantitative detection of their components [83]. This technique generates a fingerprint profile of exosomal proteins and has also been used for lipid characterization [69,83]. Gas chromatography combined with mass spectrometry (GC-MS) also plays an important role in exosome characterization: it is used for metabolite analysis in EVs [69].

MS is therefore a technique that offers high selectivity and sensitivity, enabling the identification of various types of exosomal components [69]. Furthermore, it allows for parallel monitoring and qualitative and quantitative protein analysis, significantly reducing sample complexity compared to whole biological fluids [69]. However, it requires prior sample purification and expensive instrumentation with qualified personnel [69,83]. In any case, mass spectrometry is a key tool for unraveling the protein composition of exosomes, crucial for identifying disease biomarkers [69].

#### 4.2.4. Exosomal RNA Sequencing (RNA-Seq)

In the characterization of exosomes, the detection of their nucleic acid cargo is essential. This can reflect the physiological and pathophysiological processes of the cells of origin and, in consequence, serve as biomarkers [83,86]. As indicated, exosomes transport a wide variety of nucleic acids, including protein-encoding messenger RNA (mRNA) and various forms of non-coding RNA (ncRNA), such as miRNA, long non-coding RNA (lncRNA), ribosomal RNA (rRNA), transfer RNA (tRNA), small nuclear RNA (snRNA), and circular RNA (circRNA) [83,86]. Small non-coding RNAs are the most common type of RNA found in exosomes [86]. The main challenge in the detection of exosomal RNA and DNA is their relatively low abundance per exosome, which makes most studies dependent on molecular amplification strategies [86]. Thus, the key methods used to detect nucleic acid loading are polymerase chain reaction (PCR) and its variants, such as quantitative PCR (qPCR), and RNA sequencing (RNA-seq) [83,86].

RNA-seq is a technology that uses next-generation sequencing (NGS) to assess the quantity and sequences of RNA present in a sample [86]. It is considered a fundamental tool for the proteomic characterization of exosomes and for obtaining an exosomal RNA fingerprint [86]. The typical RNA-seq workflow includes RNA extraction, reverse transcription to complementary DNA, ligation, amplification, and sequencing [83,86]. Unlike microarrays, RNA-seq is a more flexible and robust technique. It is not limited to known genomic sequences; it allows for the discovery of novel transcripts, splice variants, single-nucleotide polymorphisms, and other changes in RNA sequences [86]. It also provides both qualitative and quantitative information on the various RNA species present in a sample and can estimate RNA expression levels more accurately. RNA-seq is especially important in identifying biomarkers for diagnosis, prognosis, or therapeutic response monitoring in cancer, as it has revealed that exosomal RNA profiles differ between healthy subjects and those with various pathological conditions [86]. However, preparing libraries for RNA-seq of small RNAs can be challenging due to the formation of adapter dimers, which limits sensitivity with low sample volumes [83,86].

## 5. Methodologies for Drug Incorporation into Exosomes

Natural and biomimetic exosomes present promising features for biomedical applications, such as drug encapsulation and delivery [67,87,88,89]. As mentioned above, certain EV populations exhibit low immunogenicity, high biocompatibility and intrinsic cell tropism [67,87,89,90]. However, these properties are highly context-dependent and vary according to vesicle source, loaded cargo, administration route and recipient physiological state [23,26]. Consequently, surface engineering strategies are often employed to refine targeting precision, prolong circulation time and minimize off-target toxicity [91]. Therefore, biomimetic exosomes could have great potential as vehicles for drug delivery. For therapeutic applications, exosomes can be loaded with exogenous molecules such as small-molecule drugs that can be either hydrophobic, which tend to interact with the lipid bilayer and encapsulate within it, or hydrophilic, which encapsulate within the aqueous core (lumen) of the exosome [31,55,67].

To encapsulate the drugs, there are two main strategies: pre-loading or indirect loading and post-loading or direct loading [31,55,67,92]. In the former, the cargo is introduced into donor cells before exosome formation and isolation so that the cargo is encapsulated during natural exosome biogenesis [31,55,67]. In contrast, in the latter, the cargo is introduced into already isolated exosomes [31,55,92]. This approach is generally more effective, as it allows greater control over the encapsulation efficiency and loading capacity of the final product [31,55,67]. These strategies are applicable to both natural and biomimetic exosomes [55,67].

**Figure 6:**
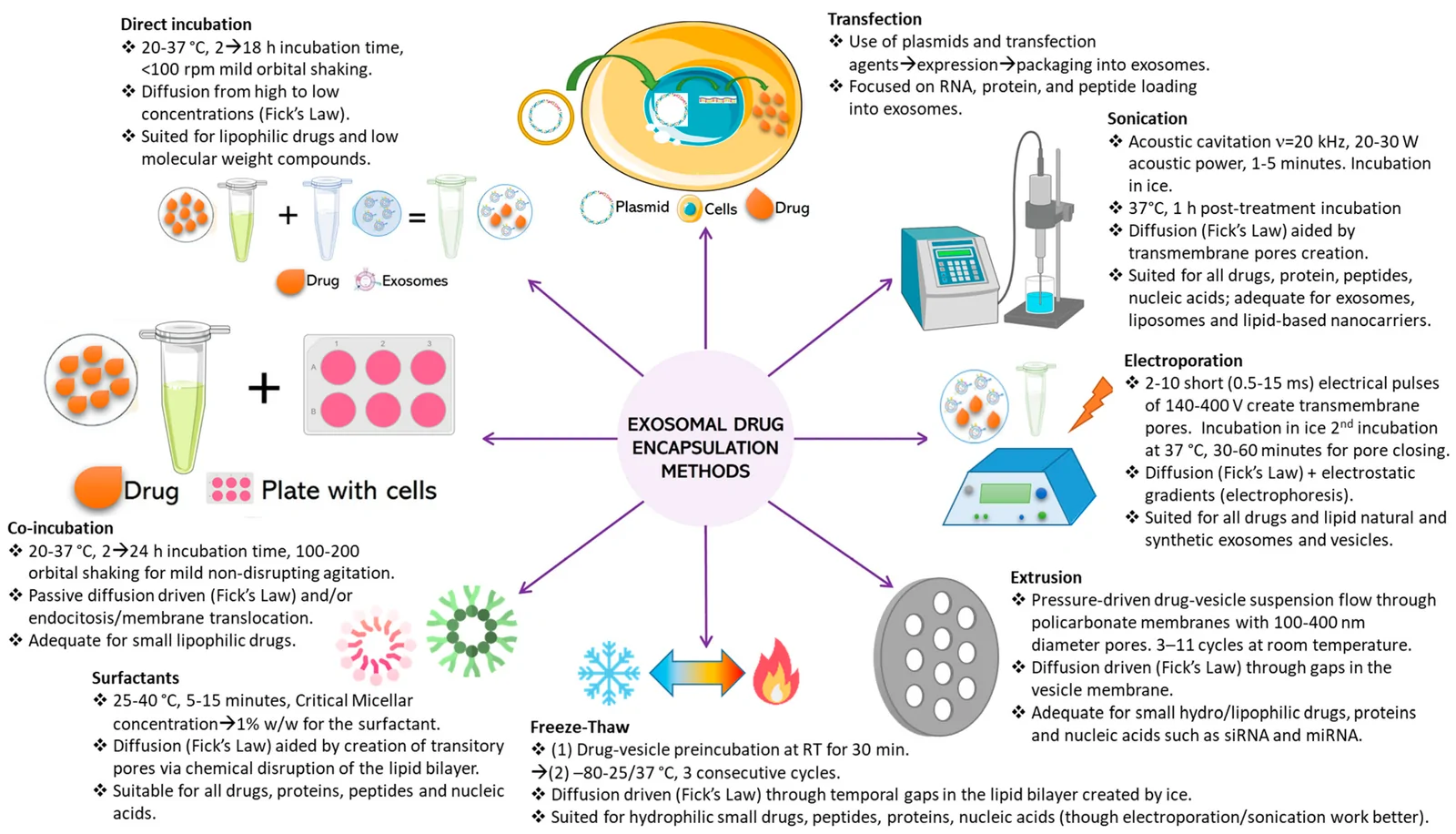
Schematic representation of the exosomal drug encapsulation methods for both natural and biomimetic exosomes. Biological methods include transfection, which involves the genetic manipulation of parental cells to modify the exosomes they secrete, and incubation, which relies on cargo diffusion across cellular membranes (indirect method: co-incubation) or directly into exosomes (direct method). Physical techniques facilitate cargo loading by disrupting membrane integrity through transient pore formation through which molecules can enter. Depending on the type of energy used to generate these pores, several techniques can be employed, including sonication (sonic cavitation), electroporation (electricity), extrusion (mechanical force), and freeze–thaw cycles (thermal energy). Chemical methods can introduce molecules into the exosome, often by temporarily altering its membrane or by exploiting its chemical properties. A notable example of this technique is the use of surfactants, which temporarily dissolve the lipids in the exosome membrane, creating transient pores.

The following subsections describe the different encapsulation methods, classified according to their nature, for a better understanding of their fundamentals [92,93]. In Figure 6 all these techniques are represented. In addition, the final subsection discusses the assessment of cargo encapsulation and key concepts related to drug loading/delivery.

### 5.1. Biological Methods

#### 5.1.1. Incubation

Drug encapsulation by incubation is a passive biological method based on the diffusion of the cargo through cellular membranes (indirect method) or exosomes (direct method), driven mainly by a concentration gradient [67,94,95]. In indirect incubation or co-incubation of the cargo with the parental cells, the desired substance is loaded into the donor cells before isolating the exosomes so that it is naturally encapsulated within them during their biogenesis and secretion by these cells [67,94,95]. This technique is especially employed to load proteins, siRNA, miRNA, chemotherapeutic agents, and other substances such as curcumin or nanomaterials [67,94,95]. Although co-incubation is a simple technique that allows for the continuous production of loaded exosomes without compromising membrane integrity, it suffers from low loading efficiency and lacks precise control over the amount of cargo incorporated [94,95]. Techniques such as low-level electrotreatment and ultraviolet irradiation have been explored to improve efficiency but are still under development [67].

Regarding the direct incubation of cargo with exosomes, the internalization of molecules occurs when the external concentration of these molecules is high, allowing the generation of a concentration gradient [94,95]. This technique is particularly suitable for hydrophobic drugs that can interact with the lipid surfaces of exosomes, such as DOX, PTX, rhodamine, and curcumin [94,95]. Despite having a hydrophilic core, the interaction of hydrophilic drugs with the lipid bilayer is limited, and passive incubation is generally less effective for these types of compounds [95]. Direct incubation has been successfully applied to encapsulate miRNAs and proteins such as brain-derived neurotrophic factor and catalase [95]. Furthermore, this technique stands out for its simplicity and its non-destructive effects on exosome integrity [94,95]. However, like co-incubation, loading efficiency is low, and loading is difficult to quantify and control due to the physicochemical properties of the molecules and exosomes [94,95]. In addition, its applicability is restricted primarily to hydrophobic drugs, and pH can influence loading efficacy [95]. Therefore, both passive incubation methods share the challenge of generally low loading efficiency compared to the active loading strategies detailed below [94,95].

#### 5.1.2. Transfection

Transfection is an active biological method characterized by being a common and stable strategy for loading nucleic acids, proteins, and peptides into exosomes [12,95]. Although they are not drugs as such, they can also have a therapeutic effect [31,95]. This method is based on the genetic manipulation of the parental cells that secrete the exosomes [12,66]. To this aim, specific plasmids are introduced (transduced) into them using transfection reagents [12,31,95]. Once transfected, the cells express the desired nucleic acids, proteins, or peptides, and these products are subsequently naturally packaged into exosomes using the donor cell’s endogenous expression and sorting machinery [31,95]. Furthermore, genetic engineering is frequently employed to express specific transmembrane proteins on the exosomal surface (engineered exosomes) [12,31,95].

This technique has been used to load miRNA-122 into exosomes derived from mesenchymal stem cells (MSCs) for the treatment of hepatocellular carcinoma. It has also been employed to load catalase mRNA into exosomes from HEK293 cells for the treatment of Parkinson’s disease [31,95]. Therefore, transfection is widely regarded as one of the most effective and stable strategies for loading nucleic acids, proteins, and peptides, ensuring high loading efficiency and facilitating their stable expression. However, it is a costly and time-consuming technique; it cannot be used to encapsulate drugs, and the use of transfection reagents can cause potential damage or contamination to cells or exosomes [12,95]. Furthermore, loading efficiency can be relatively low for certain types of cargo because intracellular sorting mechanisms are difficult to manipulate [95].

### 5.2. Physical Methods

Among the cargo encapsulation methods, physical methods, also known as active methods, stand out [67,95,96]. These include electroporation, sonication, extrusion, and freeze-thawing [67,95,96]. Physical induction involves the use of external stimuli to facilitate the incorporation of a specific cargo into exosomes by generating transient pores or mechanical tension in their membrane [67,95,96]. These simple methods can be applied to encapsulate a wide range of molecules in exosomes and can be easily applied in the laboratory [67,95]. Furthermore, they offer precise control of the process and the optimization of loading conditions [67]. In fact, optimization of encapsulation conditions and the compatibility of the molecules with the exosome membranes are essential to maintain cargo stability and prevent leakage [67]. However, these approaches may also modify the physicochemical properties of exosomes by altering their intrinsic structure [67,95,96].

#### 5.2.1. Electroporation

Electroporation is a physical method used to encapsulate various therapeutic compounds by applying an external electric field (electric pulses) to a mixture of natural or biomimetic exosomes and cargo [93,97,98]. This electric field induces the formation of small, temporary pores in the lipid bilayer of the exosome membrane, which increases its permeability, allowing compounds of interest (drugs or nucleic acids) to diffuse into the exosome [93,97,98]. Parameters such as voltage, pulse duration, and time are critical and must be optimized according to the cargo molecule and the equipment used [93,98]. After the electric pulse, the mixture is incubated (30 min or 1 h at 37 °C) to allow the exosome membrane to fully recover its integrity and the encapsulated molecules to stabilize within it [93]. Finally, purification procedures (ultracentrifugation or washing with phosphate-buffered saline) are performed to remove non-encapsulated cargo molecules and obtain the loaded and purified exosomes [93,98].

Electroporation is a particularly useful method for loading large nucleotides and, to a lesser extent, small-molecule drugs and proteins [87,98,99]. It is also a simple technique with high efficiency and molecular stability, allowing for process control by adjusting parameters [87,98,99]. However, the application of electrical pulses can damage the exosome membrane, which can lead to irreversible rupture and reduce quality and yield [87,98,99]. Furthermore, during electroporation, RNA precipitation or exosome aggregation can occur, which can reduce loading efficiency [87,98]. Finally, it is important to keep in mind that, to achieve industrial production, electroporation methods require further optimization as they can be difficult to scale [98,99].

#### 5.2.2. Sonication

Sonication is a physical encapsulation method based on the use of ultrasonic energy to temporarily disrupt the lipid bilayer of the natural or biomimetic exosome membrane, creating pores or fissures that facilitate the entry of molecules of interest [18,93,95,98,100]. Unlike electroporation, sonication involves cavitation effects and shear forces that contribute to the temporary permeabilization of the exosome membrane [95,98]. To encapsulate compounds using this technique, exosomes are mixed with the cargo and subjected to ultrasound pulses, often in an ice-water bath [93,100]. Ultrasound parameters are critical and must be optimized, including amplitude, on/off cycles, and total duration [93,100]. As with electroporation, after ultrasound treatment, the sample is incubated for 1 h at 37 °C to restore the integrity of the exosome membrane [93,100]. The sample is then centrifuged (120,000 *g* for 70 min at 4 °C) to remove the supernatant and any non-encapsulated free drug. Finally, it is washed with phosphate-buffered saline and centrifuged again to obtain the loaded exosome suspension [93,100]. It is important to note that sonication is a particularly suitable technique for loading small molecule drugs (hydrophilic and hydrophobic), but it can also be used to encapsulate proteins and nucleic acids [93,98,100]. Furthermore, it offers high loading efficiency due to the recombination of the exosomal membrane under ultrasound, which promotes drug diffusion [87,93,95,100]. Similarly, it does not require chemical or surface modification, protects the encapsulated molecules, and can produce sustained cargo release [87,98,100]. However, damage to the exosome structure and aggregation can also occur, requiring specialized instrumentation [95,98,100].

#### 5.2.3. Extrusion

Extrusion is a physical method based on the use of mechanical force to temporarily deform the exosome membrane, creating pores or inducing membrane recombination, which facilitates the entry of the cargo to be encapsulated [93,94,97]. To do this, the exosomes are mixed with the molecules to be encapsulated and passed approximately ten times through a lipid extruder containing a porous membrane with openings ranging from 100 to 400 nm [94,97]. To achieve greater efficiency, extrusion can be performed through filters with progressively smaller pore sizes, for example, 400, 200, and 100 nm [18,94,97]. Finally, as with the other methods analyzed, the sample is purified by centrifugation or filtration to remove unencapsulated cargo [93]. Beyond natural exosomes, extruding cells with specific molecules (co-extrusion) produces the synthetic exosomes described in the previous sections, ensuring enhanced productivity and scalability [12,17,18,93]. Drug concentration significantly influences the loading rate during extrusion [97].

Extrusion has proven effective for encapsulating a variety of cargoes, including small molecule drugs, nucleic acids (siRNAs), proteins, and gold nanoparticles [93,95,97]. Moreover, extrusion allows for high loading efficiency and a homogeneous population of encapsulated exosomes, making it a highly reproducible method [93,97]. However, although it is a less aggressive physical method than electroporation or sonication, it can still damage the exosome membrane, affecting properties such as the zeta potential and the structure of membrane proteins [93,95,97,99]. For example, it has been reported that exosomes loaded with porphyrins by extrusion were cytotoxic, unlike those loaded using other techniques [97]. Furthermore, this technique cannot be applied to all exosome types, since some cells secrete unstable exosomes not suitable for extrusion [93].

#### 5.2.4. Freeze–Thaw

To encapsulate natural and biomimetic exosomes (hybrid exosomes), freeze–thaw cycles can also be performed, since freezing causes the water inside them to expand, generating temporary fissures or pores in the exosomal membrane [68,93]. As the temperature of the suspension increases (thawing), the ice melts, and water, along with the molecules of interest, enters the exosomes through these pores [93]. As the expansion tension decreases, the pores of the exosomal membrane contract, thus encapsulating the drug inside. The process can also induce recombination of the exosomal membrane [12,93,94]. To carry out this physical method, it is necessary to freeze the mixture of exosomes with the compound of interest at −80 °C or in liquid nitrogen. Afterwards, it is thawed at room temperature or at 37 °C, and the freeze–thaw cycle is repeated at least three times to promote better encapsulation of the drug [68,93,101]. Encapsulation efficiency may depend on the number of repetitive cycles. It is to be emphasized that the cargoes best encapsulated with this method are water-soluble drugs, low molecular weight compounds, and nucleic acids such as DNA or RNA [93]. Therefore, this method does not damage the exosome structure, prevents drug degradation, and can be used to fuse these EVs with liposomes, thus creating hybrid exosomes with functional lipids and membrane proteins that can improve cellular uptake [12,68,93,94]. Despite these advantages, exosome stability can be compromised, production efficiency is low, exosomes can aggregate, and loading efficiency is lower than that of other physical treatments such as sonication and extrusion [68,87,93,95,101].

### 5.3. Chemical Methods

Chemical methods can introduce diverse compounds into the exosomes, often by temporarily altering their membrane or taking advantage of chemical properties [95,96,98]. This technique includes the use of surfactants such as saponin, which temporarily dissolve the lipids in the exosome membrane, creating transient pores through which molecules can enter [67,92]. Lipofectamine, a transfection reagent capable of forming lipid complexes with these molecules, is also used to encapsulate primarily nucleic acids [67,98]. These complexes can then be incubated with the exosomes to facilitate cargo entry [46]. Another example of a chemical method is the formation of ion pairs as a result of the interaction of opposite charges [96,98]. In this technique, ionic drugs can form ion pairs with ions present in the exosome membrane, for example, doxorubicin with phosphatidylserine [98]. In addition, in situ synthesis is possible, which implies that chemical reactions occur directly within or on the exosomes to synthesize nanomaterials [95,96]. It is a widely used method for loading metal nanoparticles, although it is a complex process with multiple technological challenges [95,96]. Finally, chemical methods can be used to modify the surface of exosomes (engineered exosomes), aiming to modify their physicochemical properties and improve delivery to target cells [66,95,96,98].

### 5.4. Assessment of Cargo Encapsulation

Accurately assessing cargo loading is fundamental in EV-based drug delivery [102,103]. To distinguish encapsulated from non-encapsulated material, EV preparations require thorough purification, especially after applying direct loading protocols [103]. Unpurified samples often retain free cargo, aggregates, or surface-adsorbed molecules that contribute to false-positive loading signals and overestimation [103]. Selecting among purification techniques—such as SEC, ultrafiltration, dialysis, or density-gradient centrifugation—depends on the specific physicochemical properties of the cargo and EVs [103]. Accordingly, the efficiency of the chosen purification procedure should ideally be assessed by quantifying both EV-associated and free-cargo fractions. Importantly, even when rigorous purification methods are applied, the resulting preparation may still consist of a heterogeneous mixture of cargo-loaded and empty EVs [103,104].

Following the removal of non-encapsulated material, cargo loading is typically evaluated using two distinct metrics: encapsulation efficiency (EE) and loading capacity (LC), which provide complementary rather than interchangeable information [105]. EE reflects the proportion of total input drug that becomes encapsulated, calculated as the ratio of encapsulated drug mass to the initial drug input [106,107]. In contrast, LC measures the mass of loaded cargo relative to particle quantity, routinely reported normalized to total EV protein (weight/weight ratio) or particle count (weight/particle ratio) [106,107]. While EE serves as a key parameter for assessing economic and process efficiency by accounting for non-encapsulated drug loss [107], LC directly dictates carrier dosing; overly low LC values demand excessive EV doses that may compromise therapeutic viability [107]. Because cargo physicochemical properties and EV matrix characteristics substantially influence these parameters, reported EE and LC values should always be contextualized with the corresponding purification protocol, experimental conditions, and normalization strategy [105,106].

Cargo detection in purified EVs does not by itself demonstrate luminal internalization [105,106]. Distinguishing luminal loading from surface adsorption is critical, as the physicochemical properties of the cargo can influence whether it is retained within the vesicle lumen, associated with the lipid bilayer, or exposed at the EV surface [105,106]. This topological distinction is important to avoid overestimating loading metrics and to better understand the subsequent delivery mechanism [105,106,108]. To confirm true luminal encapsulation over surface-bound material, international guidelines such as MISEV2018 [108] recommend enzymatic protection assays by comparing intact versus detergent-treated vesicles. However, analytical cargo detection does not inherently guarantee structural integrity or bioactivity [105]. Functional assays should therefore complement quantitative characterization to confirm therapeutic potency [105,109].

Moreover, cargo encapsulation must be distinguished from functional intracellular delivery [110,111]. Since endocytosis represents the primary route of EV entry, cellular internalization does not guarantee functionality, as cargo can be degraded during endo-lysosomal maturation [111]. Specialized approaches—such as cytosolic reporter assays and subcellular fractionation—have been developed to differentiate EV uptake from true cytosolic delivery [106]. Finally, EV recovery must also be considered when evaluating loading performance, as loading procedures can alter vesicle yield and skew apparent metrics [103,106]. Despite its importance, the lack of quantitative tracking of EV amounts pre- and post-loading remains a widespread limitation in the literature [103]. Accordingly, standardization frameworks strongly advocate quantifying particle numbers before and after loading to accurately determine EV loss, rather than relying on protein assays that correlate poorly with particle counts [103,106].

Overall, current evidence demonstrates that no single EV loading method is universally superior [105,106,109]. The performance of any given strategy depends heavily on cargo properties, EV source and composition, specific loading conditions, purification protocols, and the metrics used to quantify loading [104,105,106,109]. Consequently, evaluating loading approaches should extend beyond EE or LC alone to incorporate cargo recovery, luminal localization, cargo integrity and bioactivity, EV yield, and functional intracellular delivery [104,105,106,109]. Adopting a standardized, multidimensional evaluation framework will facilitate rigorous cross-study comparisons and advance loading strategies that achieve efficient incorporation without compromising EV integrity or therapeutic potency [104,105,106,109].

## 6. Future Perspectives and Conclusions

Natural exosomes represent a highly promising frontier in nanomedicine and healthcare applications. This is due to their biocompatibility, low immunogenicity, and unique capability to penetrate complex biological barriers, apart from the fact that they are able to transport complex cargo, as they can naturally sort and package fragile molecules, like mRNA, siRNA, and proteins, protecting them from degradation in the body. Nevertheless, these properties are context-dependent (cellular origin, structural properties, processing methodologies, etc.) and it is necessary to improve targeting and clearance by the immune system through different strategies such as surface engineering [23,24,26]. Moreover, several drawbacks are still to be addressed: (i) the large-scale clinical application of natural vesicles—despite their intrinsic homing capability and biological integrity—is severely hindered by inherently low production yields, cargo heterogeneity, and the technical challenges associated with conventional isolation methods that usually lead to high batch-to-batch variability; (ii) the innate structural and compositional heterogeneity of exosomes, which hinders scale-up and jeopardizes regulatory issues key to their clinical application; (iii) instability (or high trend to degradation), often requiring extreme storage conditions (such as −80 °C) to maintain their structural integrity, again complicating their final use at the clinical level, and (iv) safety risks due to their animal or human origin, which advocates for extensive purification to pass regulatory constraints before clinical application [55,93].

To overcome the drawbacks of natural exosomes, biomimetic exosomes have emerged as a robust solution because they offer up to a 100-fold increase in industrial scalability and a more precise control over their physical structure and chemical and functional composition [18,55,112]. These nanoparticles can easily be manipulated, adjusting their average size and size distribution, their shape, and the composition of their membrane, even including surface ligands to fine-tune drug release rates. Concurrently, they meet the stringent demands of the pharmaceutical industry for pure, standardized, and large-scale manufacturing [12,18,67]. However, their relative simplicity both in structure and composition creates a number of drawbacks: (i) they have a lower targeting specificity, as their surface is simpler, lacking the complex array of proteins and ligands natural to exosomes, so these nanoparticles need further surface engineering to try to overcome this restriction and target specific tissues precisely; (ii) their lack of CD47 protein results in a higher immune clearance, as the nanoparticles recognized by white blood cells and cleared from the body; (iii) they trend to accumulate in the hepatobiliary system and be eliminated through the duodenum; the slow blood flow in the liver sinusoids allows only the smallest nanoparticles to enter, and the natural coating of nanoparticles with certain proteins, such as apolipoprotein E, allows for their capture by Kupffer cells, which phagocytose them, and hepatocytes, which direct them into the gallbladder.

To avoid the aforementioned drawbacks, while profiting from the advantages of natural exosomes, synthetic exosome complexity has been increased by further, more complex surface engineering, and methods to create hybrid exosomes by fusing natural exosomes with synthetic exosomes have been developed. Engineering synthetic exosomes includes the modification of their surface by PEGylation, human serum albumin (HSA), certain cell-penetrating peptides, some polysaccharides and glycans, such as chitosan, dextran, or hyaluronic acid, and several zwitterionic and hydrophilic polymers: polysarcosine, poly(carboxybetaine), and poly(2-oxazoline)s [113]. The fusion of natural cell membranes, such as erythrocytes, platelets, and macrophages, is also an alternative similar to the construction of hybrid exosomes. These hybrid nanoparticles, in comparison to their purely synthetic counterparts, have superior biocompatibility, enhanced drug capacity—maintaining control of drug loading, targeted delivery, and prolonged circulation, avoiding immune clearance [12]. Still, they are far from being a complete solution nowadays, as they still suffer from complex manufacturing (with the problems present in exosome production batches), physical damage due to the fusing protocols (affecting the delicate exosomal lipid bilayer, leading to cargo leakage or reduced cellular uptake), low yield and scalability, and, in consequence, regulatory challenges not different from those faced by natural exosomes and their biomimetic synthetic exosome-like nanoparticles.

Therefore, and prior to the successful clinical translation of these platforms, whether natural, synthetic, or hybrid, key challenges regarding safety, a deeper evaluation of potential immune responses, and the optimization of production methodologies must be resolved [68,93]. Thus, ensuring therapeutic efficacy requires standardized characterization methods to validate particle size via NTA, as well as purity through universal protein markers, including tetraspanins (CD9, CD63, CD81) and cytosolic proteins (Alix or TSG101) [71,89]. Furthermore, the choice of drug encapsulation strategies must carefully balance the high loading efficiency of active methods (electroporation, sonication, or extrusion) with the preservation of structural membrane integrity offered by passive or chemical approaches [68,95,97]. Ultimately, the future of these nanoparticles relies on establishing a global consensus on quality control parameters and strict GMP-compliant protocols, ensuring the safety, stability, and standardization required to revolutionize personalized therapies [67,68,88]. The starting point has been the Minimal Information for Studies of Extracellular Vesicles (MISEV) developed by the International Society for Extracellular Vesicles (ISEV) [114].

## Figures and Tables

**Table 1:** Therapeutic research examples of using exosomes and exosome biomimetics (either hybrid or synthetic) for cancer [27,28,29,30,31].

Disease	Type of Exosome	Exosome Source	Isolation Methods/ Synthesis Method	Characterization Methods	Load	Encapsulation Method	Disease Model	Therapeutic Effect	Reference
Colorectal and cervical cancer	Natural exosome	Curcumae Rhizoma	Differential ultracentrifugation + density gradient ultracentrifugation	TEM DLS RNA-seq UPLC-MS/MS	Formononetin Astragaloside IV Calycosin	Sonication	In vitro (Caco-2 and HeLa) In vivo (rats)	Improved adsorption, bioavailability, and antitumor effect	[27]
Colorectal cancer	Natural exosome	Human cord blood mesenchymal stem cells	Differential ultracentrifugation	TEM FCM	Anti-miRNA-221	Electroporation	In vitro (Caco-2 and HCT116) In vivo (rats)	Inhibition of tumor cell growth	[28]
Colorectal cancer	Hybrid exosome	Neutrophil membrane + liposome	Neutrosomes: Freeze–thaw cycles + sonication + differential ultracentrifugation + extrusion Liposomes@LAPA: thin-film dispersion method	TEM DLS FCM	β-Lapachone (LAPA)	Fusion of neutrosomes and liposomes@LAPA: sonication + extrusion	In vitro (CT-26) In vivo (BALB/c male mice)	Potent immunogenic cell death induction	[29]
Lung and colorectal cancer	Engineered exosome	Commercial bovine milk	Ultracentrifugation + chemical modification of exosomes to express folate on the surface	TEM DLS Western blot	Doxorubicin	Direct incubation	In vitro (A549 and HCT116) In vivo (rats)	Targeted delivery to tumors and inhibition of tumor growth	[30]
Colorectal cancer	Engineered exosomes	HEK293T cells	Previous transfection of the cells to express Her2-affibody + ultracentrifugation	TEM DLS Western blot	5-Fluorouracil miR-21 inhibitor oligonucleotide	Electroporation	In vitro (HCT-1165FR) In vivo (BALB/c female mice)	Arrest of the cell cycle and reduction in tumour proliferation	[31]

**Table 2:** Examples of regenerative medicine approaches promoted by natural, synthetic or hybrid exosomes [32,33,34,35,36].

Disease	Type of Exosome	Exosome Source	Isolation Methods/ Synthesis Method	Characterization Methods	Load	Encapsulation Method	Disease Model	Therapeutic Effect	Reference
Bone defect	Natural exosome	Human adipose-derived mesenchymal stem cells	Ultracentrifugation	TEM NTA Western blot	miRNA-375	Transfection	In vitro (human bone marrow stromal cells) In vivo (rats)	Increased bone regeneration	[32]
Skin photoaging	Natural exosome	Adipose-derived stem cells	Ultracentrifugation + precipitation with commercial kit	TEM nFCM Western blot	miRNA-1246	Transfection	In vitro (human skin fibroblasts) In vivo (rats)	Protection against photoaging	[33]
Hair loss	Natural exosome	Dermal papilla cells	Precipitation with commercial kit	TEM NTA Western blot	miRNA-181-5p	Transfection	In vitro (dermal papilla cells and hair follicle stem cells)	Increased growth and development of the hair follicle	[34]
Osteoporosis	Synthetic exosomes	NIH-3T3 cells	Cell membranes: membrane and protein extraction with commercial kit ACP/CP: coprecipitation method	TEM DLS Western blot	Amorphous calcium phosphate (ACP) Creatine phosphate (CP)	Sonication	In vitro (human umbilical vein endothelial cells) In vivo (BALB/c female mice)	Significant promotion of osteoporotic bone recovery	[35]
Liver injury and ischemia	Synthetic exosomes	Mesenchymal stem cells	Priming of the cells with inflammatory cytokines + one-step extrusion (isolation and encapsulation)	TEM FCM Western blot LC-MS	Secretome of the mesenchymal stem cells	One-step extrusion	In vitro (endothelial cells and hepatic stellate cells) In vivo (rats)	Pro-angiogenic, pro-proliferative and anti- inflammatory effects	[36]

**Table 3:** Therapeutic approaches to ocular diseases based on the application of natural or biomimetic exosomes [37,38,39,40,41].

Disease	Type of Exosome	Exosome Source	Isolation methods/ Synthesis Method	Characterization Methods	Load	Encapsulation Method	Disease Model	Therapeutic Effect	Reference
Age-related macular degeneration	Natural exosome	Commercial bovine milk	Differential ultracentrifugation	TEM NTA DLS	Curcumin	Sonication	In vitro (ARPE-19)	Antioxidant potential	[37]
Diabetic retinopathy	Natural exosome	Human umbilical cord-derived mesenchymal stem cells	Differential ultracentrifugation	TEM NTA Western blot	miRNA-126	Transfection	In vitro (human retinal endothelial cells) In vivo (rats)	Reduction in retinal inflammation	[38]
Glaucoma	Natural exosome	Human adipose-derived mesenchymal stem cells	Tangential flow filtration	TEM NTA Western blot qPCR	Nicotinamide	Extrusion	Ex vivo (mouse retinal explant) In vivo (rabbits)	Retinal ganglion cells neuroprotection	[39]
Cataract	Hybrid exosomes	Human lens epithelial cells + liposomes	Exosomes: N/A Liposomes@Cur (LS@Cur): thin-film hydration method LSP@Cur: amide coupling to graft PENE into LS@Cur	TEM DLS NTA Western blot	Curcumin (Cur)	Fusion of exosomes and LSP@Cur: sonication + extrusion	In vitro (human lens and corneal epithelial cells) In vivo (SD rats)	Effective prevention of cataract formation	[40]
Dry eye	Hybrid exosomes	Human and mouse corneal epithelium cells + liposomes	Exosomes: differential ultracentrifugation Liposomes-siRNA (siRNA-Lipo): thin-film hydration method + extrusion	TEM NTA FCM RNA-seq qPCR	siRNA (NF-κB inhibitor zeta)	Fusion of exosomes and siRNA-Lipo: DNA zipper-mediated membrane fusion	In vitro (HCECs and RAW264.7 macrophages) In vivo (rats)	Promotes tear secretion and restores corneal integrity	[41]

**Table 4:** Comparison of exosome isolation methods [8,43,48,49,61,62,63,64].

Method	Recovery	Purity	Drug Delivery Potential	Process Time	Batch Reproducibility	Cost (Equipment/Consumables)	Application Field	References
Ultracentrifugation	Low	Medium	Low	Long (4–6 h)	Low	High/Low	Analytical-scale isolation	[49,61,62]
Ultrafiltration	Medium	High	Medium	Medium (30–90 min)	Medium–high	Low/Low	Analytical-scale concentration and small-batch processing	[61,63]
Tangential Flow Filtration	High	High	High	Medium (1–2 h)	Very high	High/Very low	Manufacturing-scale purification (GMP)	[48,63]
Size-exclusion chromatography	High	High	High	Short (20–30 min)	High	Low/Medium	Analytical and pilot-scale	[8,62,63]
Precipitation	High	Low	Very low	Short-medium (30 min–2 h)	Medium	Low/High	Analytical-scale screening	[49,61,62]
Immunoaffinity	Medium	High	Low	Long (2–4 h)	High	Very high	Analytical-scale diagnostics	[49,61]
Microfluidics	Medium- high	High	High	Short to long (5 min–10 h)	High	High/Medium–high	Analytical-scale isolation and point-of-care diagnostics	[43,64]
Tandem TFF and SEC	High	High	High	Medium (2–3 h)	Very high	Medium–high/Medium	Advanced manufacturing-scale and clinical production	[49,63,64]

## Data Availability

The original contributions presented in the study are included in the article, further inquiries can be directed to the corresponding authors.
